# Biomarker identification for Alzheimer’s disease through integration of comprehensive Mendelian randomization and proteomics data

**DOI:** 10.1186/s12967-025-06317-5

**Published:** 2025-03-06

**Authors:** Hui Zhan, Davis Cammann, Jeffrey L. Cummings, Xianjun Dong, Jingchun Chen

**Affiliations:** 1 Interdisciplinary Neuroscience Program, University of Nevada, Las Vegas (UNLV), Las Vegas, NV USA; 2 Nevada Institute of Personalized Medicine, University of Nevada, Las Vegas (UNLV), Las Vegas, NV USA; 3 School of Life Science, University of Nevada, Las Vegas (UNLV), Las Vegas, NV USA; 4 Chambers-Grundy Center for Transformative Neuroscience, Department of Brain Health, Kirk Kerkorian School of Medicine, University of Nevada, Las Vegas (UNLV), Las Vegas, NV USA; 5 Stephen and Denise Adams Center for Parkinson’s Disease Research, Yale School of Medicine, Yale University, New Haven, CT USA; 6 Department of Neurology and Section of Biomedical Informatics and Data Science (BIDS), Yale School of Medicine, Yale University, New Haven, CT USA

## Abstract

**Background:**

Alzheimer’s disease (AD) is the main cause of dementia with few effective therapies. We aimed to identify potential plasma biomarkers or drug targets for AD by investigating the causal association between plasma proteins and AD by integrating comprehensive Mendelian randomization (MR) and multi-omics data.

**Methods:**

Using two-sample MR, *cis* protein quantitative trait loci (*cis*-pQTLs) for 1,916 plasma proteins were used as an exposure to infer their causal effect on AD liability in individuals of European ancestry, with two large-scale AD genome-wide association study (GWAS) datasets as the outcome for discovery and replication. Significant causal relationships were validated by sensitivity analyses, reverse MR analysis, and Bayesian colocalization analysis. Additionally, we investigated the causal associations at the transcriptional level with *cis* gene expression quantitative trait loci (*cis*-eQTLs) data across brain tissues and blood in European ancestry populations, as well as causal plasma proteins in African ancestry populations.

**Results:**

In those of European ancestry, the genetically predicted levels of five plasma proteins (BLNK, CD2AP, GRN, PILRA, and PILRB) were causally associated with AD. Among these five proteins, GRN was protective against AD, while the rest were risk factors. Consistent causal effects were found in the brain for *cis*-eQTLs of *GRN*, *BLNK*, and *CD2AP*, while the same was true for *PILRA* in the blood. None of the plasma proteins were significantly associated with AD in persons of African ancestry.

**Conclusions:**

Comprehensive MR analyses with multi-omics data identified five plasma proteins that had causal effects on AD, highlighting potential biomarkers or drug targets for better diagnosis and treatment for AD.

**Supplementary Information:**

The online version contains supplementary material available at 10.1186/s12967-025-06317-5.

## Introduction

Alzheimer’s disease (AD) is a progressive neurodegenerative disorder, characterized by abnormal accumulation of the amyloid-beta (Aβ) plaques and tau tangles in the brain [1]. Recent progress in AD therapies mainly includes disease-modifying biologic therapy focused on reducing amyloid plaque to slow cognitive decline and advances in neuropsychiatric symptom treatment by ameliorating agitation [2]. The core biomarkers of AD neuropathologic change mainly include imaging and cerebrospinal fluid (CSF) or plasma levels of Aβ, phosphorylated tau (p-tau), and tau [3]. Some plasma p-tau 217 assays have exhibited equivalent accuracy to approved diagnostic CSF assays [4, 5]. Compared to imaging and CSF biomarkers, blood-based biomarkers are less invasive and more cost-effective; they have the potential to act as screening tests, diagnostic tests, prognostic assessments, or monitoring tests [6]. Identifying blood-based biomarkers involved in the AD biological process is essential for comprehensively understanding disease progression and developing a precision therapeutic strategy.

AD is a complex and multifactorial condition influenced by many risk factors, including genetic, behavioral, environmental, lifestyle, and medical factors, among which genetic risk factors play a crucial role due to the high heritability of AD [7, 8]. With increasing large-scale genome-wide association studies (GWASs), more risk loci have been reported to be associated with AD. These studies help to decipher the underlying pathophysiological mechanisms of AD and identify potential biomarkers and drug targets [9, 10]. However, most single nucleotide polymorphisms (SNPs) identified by GWASs are located in non-coding genomic regions, and their function is largely unknown. With advanced large-scale and high-throughput proteomics techniques, proteomics data from diverse populations are increasingly integrated with systems genetics to identify protein quantitative trait loci (pQTLs) — genetic variants associated with altered protein abundance [11, 12]. By integrating multi-omics data (GWAS data and pQTL data) and Mendelian randomization (MR), we have the tools to explore the causal relationships between protein abundance and disease phenotype [13]. Mimicking the random assignment of treatment in randomized controlled trials, MR uses Mendel’s second law of independent segregation of genetic alleles to infer the potential reliable causal relationship between a risk factor (exposure) and disease risk (outcome) [14]. To date, the integration of proteome-wide and transcriptome-wide MR studies has reported potential biomarkers or drug targets for AD, chronic kidney disease, depression, Parkinson’s disease, and stroke [15–17]. With expanded coverage of antibody-based Olink assays, large proteomic datasets became available from different ancestries, where approximately twentyfold more plasma pQTLs were identified than found through previous antibody-based studies [18]. The large-scale plasma pQTL data are invaluable to identifying candidate plasma biomarkers or therapeutic targets for many complex disorders like AD. Previous studies have leveraged longitudinal proteomics data from the UK Biobank to develop predictive models for incident AD and all-cause dementia, with the same plasma pQTL data providing genetic support for those proteins as early biomarkers and potential therapeutic targets [19, 20]. Although these studies have provided valuable insights into AD, leveraging updated datasets and refined analytical approaches is crucial to uncover novel plasma proteins, especially explore ancestry-specific associations, and better understand the protein-AD relationships at the transcriptional level.

In this study, we aimed to identify plasma proteins that are causally associated with AD, with the intent of identifying potential plasma biomarkers or drug targets for AD. Leveraging the largest European ancestry plasma pQTL dataset and AD GWAS summary statistics, we conducted primary MR analysis followed by robust sensitivity analyses for discovery data. We then did replication, Bayesian colocalization analysis and phenotype scanning to validate our results from the discovery data. Additional replication at the transcriptional level was also used for validation. Finally, MR analysis was conducted between plasma pQTL and AD in data derived from individuals of African ancestry.

## Methods

### Study design

**Fig. 1:**
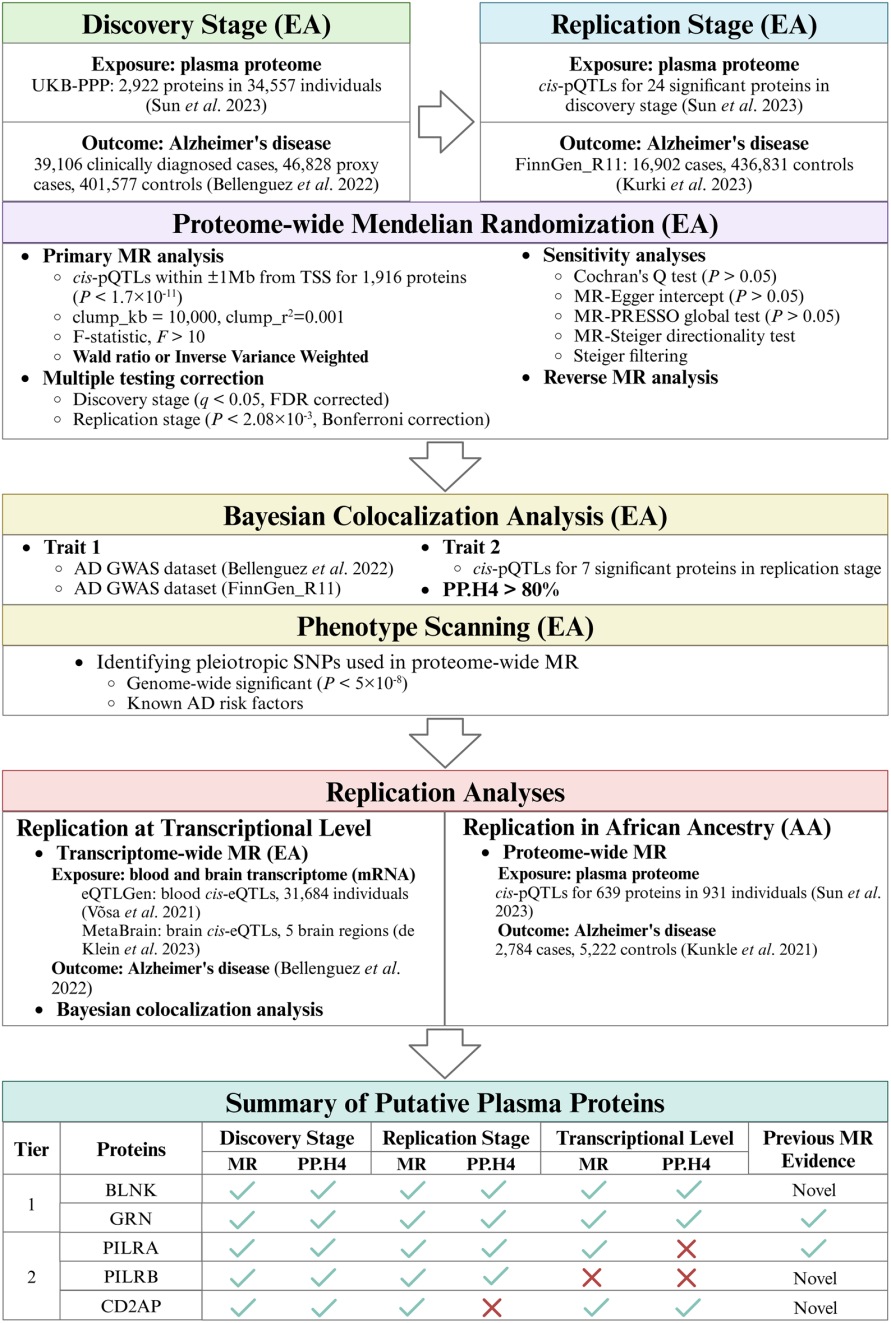
Overview of the study design. EA, European ancestry; UKB-PPP, UK biobank Pharma Proteomics Project; MR, Mendelian randomization; *cis*-pQTLs, cis protein quantitative trait loci within ± 1 Mb from TSS of the protein-coding gene; GWAS, genome-wide association study; Mb, megabase; TSS, translation start site; FDR, false discovery rate; PP.H4, posterior possibility of hypothesis 4; SNP, single nucleotide polymorphisms; *cis*-eQTLs, cis gene expression quantitative trait loci within ± 1 Mb from TSS of protein-coding gene; mRNA, messenger RNA; AA, African ancestry

To ensure unbiased and robust MR results, we conducted MR analysis following the STROBE-MR checklist (https://www.strobe-mr.org/) [21]. Figure 1 provides a comprehensive overview of the study design. Specifically, our study included the following steps. (i) In the discovery stage, we attempted to identify the potential causal associations between genetically predicted plasma protein abundance and AD in those with European ancestry. (ii) In the replication stage, we conducted replication for the potential causal plasma proteins from the discovery stage in another independent AD GWAS dataset, the Finnish cohort (FinnGen_R11). (iii) We performed Bayesian colocalization analysis to confirm shared causal variants between two traits and conducted phenotype scanning to investigate potential horizontal pleiotropy. (iv) We conducted replication analyses to further support the putative casual associations with blood and brain transcriptome data. (v) The causal associations were explored in individuals of African ancestry.

### Data sources

In this study, the *cis*-pQTLs or *cis* expression quantitative trait loci (*cis*-eQTLs) were defined as an SNP within 1 megabase (Mb) upstream or downstream from the transcription start site (TSS) of the protein-coding gene. Using the Olink platform, Sun et al. identified 14,287 primary genetic associations for 2,922 plasma proteins in 34,557 individuals of European ancestry from the UK Biobank Pharma Proteomics Project (UKB-PPP) [18]. For exposure data in two-sample MR, we leveraged the *cis*-pQTLs on autosomes of 1,916 plasma proteins from Sun et al.’s study to extract genetic variants as instrumental variables (IVs). In the discovery stage, the outcome data came from the discovery data of Bellenguez et al.’s study, including 39,106 clinically diagnosed persons with AD, 46,828 ‘proxy’ AD cases (based on a questionnaire of parental disease history), and 401,577 controls [9]. In the replication stage, the outcome data for AD was obtained from the FinnGen Release 11 (FinnGen_R11, https://www.finngen.fi/en, Phenocode: G6_AD_wide), including 16,902 cases and 436,831 controls [22]. In FinnGen, the AD cases under Phenocode G6_AD_wide are determined by a combination of hospital records, mortality data, and medication use. Next, blood *cis*-eQTLs and brain *cis*-eQTLs were used as exposure for replication analyses at the transcriptional level. The blood *cis*-eQTLs were downloaded from eQTLGen Consortium Phase 1 (https://eqtlgen.org/cis-eqtls.html), including 19,250 genes from 31,684 individuals [23]. The majority of individuals were of European ancestry in this study. The *cis*-eQTLs of 5 brain regions were from MetaBrain (https://www.metabrain.nl/cis-eqtls.html), including cortex (2,683 individuals), basal ganglia (208 individuals), hippocampus (168 individuals), cerebellum (492 individuals), and spinal cord (108 individuals) [24]. All individuals were of European ancestry. Finally, the causal effect was also explored in those with African ancestry. The exposures were autosomal *cis*-pQTLs in African ancestry from the same study as used for the European ancestry studies, where 639 proteins were identified in 931 individuals of African ancestry [18]. AD GWAS data from 2,784 AD cases (including both autopsy- and clinically-confirmed) and 5,222 controls of African ancestry was used as the outcome [25]. Information on Aβ pathology status was unknown for AD cases across all three AD GWAS datasets used in this study.

All summary statistics utilized in this study were obtained from publicly available databases. The details of all datasets were summarized in Additional File 1: Table S1.

### Two-sample Mendelian randomization analysis

Two-sample MR was performed to estimate the causal effect of the plasma protein level on AD using the “TwoSampleMR” R package (https://github.com/MRCIEU/TwoSampleMR, v0.6.2) [26]. To generate strong independent genetic IVs from plasma proteins, three criteria were used for IV selection: (a) SNPs within ± 1 Mb of TSS and with *P* < 1.7 × 10^− 11^ (the significant threshold obtained from original paper with Bonferroni correction) on autosomes were selected for primary MR analyses; (b) SNPs in each *cis*-pQTL were clumped locally with default parameters (clump_kb = 10,000, clump_r^2^ = 0.001) using the European population in the 1000 Genomes Project as reference [27, 28]; (c) only SNPs with an F-statistic > 10 were selected to filter out weak genetic IVs, following the calculation method outlined by Chen et al. [29, 30]. This approach ensures that the selected SNPs are robust and relevant for the MR analysis, thereby strengthening the conclusions drawn from the genetic data. The Wald ratio method was used to calculate the causal estimate when only one SNP remained after harmonization [31]. The inverse-variance weighted (IVW) method was used to obtain the causal estimate when multiple SNPs were kept after harmonization [32]. MR-Egger and weighted median methods were also used to estimate the causal effect of exposure on outcome with multiple genetic IVs [33, 34]. To ensure robustness in MR estimates and minimize bias from pleiotropy, the causal effect directions inferred from IVW, MR-Egger, and Weighted Median methods should be consistent. The Cochran’s Q test was conducted to detect heterogeneity in the IVW estimate, with a p-value less than 0.05 indicating significant heterogeneity [35, 36]. The intercept of MR-Egger regression was used to account for horizontal pleiotropy, with a p-value less than 0.05 suggesting significant evidence of horizontal pleiotropy [34, 37]. The MR-PRESSO test was performed to identify horizontal pleiotropic outliers, and a p-value of the global test less than 0.05 implied significant evidence of horizontal pleiotropy [38]. Steiger filtering was applied to validate the selected genetic IVs, ensuring they explain more variance in the exposure (plasma protein level) than in the outcome (AD). Additionally, the MR-Steiger directionality test was performed to confirm the true causal direction of plasma protein level on AD [39]. Reverse MR analysis was performed to investigate the potential effect of AD on plasma protein levels. The same IV selection criteria used for plasma *cis*-pQTLs were applied to the AD GWAS data, and the same sensitivity analysis methods (e.g., Cochran’s Q test, MR-Egger intercept) were used to ensure consistency and robustness [40]. For the primary MR analyses, the false discovery rate (FDR) method was employed for multiple testing correction, and the FDR-corrected p-value (*q*) of less than 0.05 for the Wald ratio or IVW method was adopted as the threshold for significant causal estimates in the discovery stage.

In the replication stage, the same criteria for IV screening used in the discovery stage were applied to validate significant causal plasma proteins, along with the same sensitivity analyses. For multiple testing corrections, MR associations were considered statistically significant at *P* < 2.08 × 10^− 3^ (0.05/24 with Bonferroni correction).

The additional transcriptome-wide MR analysis for the replication at the transcriptional level was conducted using the same methods as in the discovery stage, followed by Bayesian colocalization analysis (see below). SNPs in each *cis*-eQTL for the validated protein-coding genes were screened at genome-wide significant threshold (*P* < 5 × 10^− 8^) and clumped using the same method in the discovery stage. The transcriptome-wide MR associations were considered statistically significant at *P* < 7.14 × 10^− 3^ (0.05/7 with Bonferroni correction).

The additional proteome-wide MR analysis for those of African ancestry was performed following the same MR analysis pipeline in the discovery stage. Genetic IVs were selected for 639 plasma *cis*-pQTLs (*P* < 1.7 × 10^− 11^, adopted from original paper with Bonferroni correction) and locally clumped with the African population in the 1000 Genomes Project as a reference [27, 28]. MR associations were considered statistically significant at *q* < 0.05 (FDR corrected).

### Bayesian colocalization analysis

To determine if the causal associations were driven by shared causal variants, we conducted Bayesian colocalization analysis for plasma proteins identified by proteome-wide MR in both the discovery stage and replication stage. Bayesian colocalization analysis was conducted using the “coloc” R package (https://chr1swallace.github.io/coloc, v5.2.3) to test the five hypotheses, including hypothesis 0 (neither trait has a genetic association in the region), hypothesis 1 (only trait 1 has a genetic association in the region), hypothesis 2 (only trait 2 has a genetic association in the region), hypothesis 3 (both traits are associated, but with different causal variants), and hypothesis 4 (both traits are associated and share a single causal variant) [41, 42]. Bayesian colocalization analysis was performed to evaluate the posterior probability of those five hypotheses with prior probabilities set as P_1_ = 1 × 10^− 4^, P_2_ = 1 × 10^− 4^, and P_12_ = 1 × 10^− 5^ [42]. The summary statistics for AD in the discovery and replication stages were used as trait 1, separately. All SNPs in *cis*-pQTLs of each significant protein in the replication stage were used as trait 2. For subsequent colocalization analysis following the transcriptome-wide MR analysis, the AD GWAS dataset in the discovery stage was used as trait 1 and the *cis*-eQTLs of each protein-coding gene were used as trait 2. The posterior probability of hypothesis 3 (PP.H_3_) greater than 80% suggests the association is mainly influenced by horizontal pleiotropy. The posterior probability of hypothesis 4 (PP.H_4_) greater than 80% indicates their causal effects were likely driven by shared variants. The same parameters and thresholds above were applied to all colocalization analyses in this study. The “locusplotr” R package (https://mglev1n.github.io/locusplotr, v0.5.0) was used to generate regional association plots for colocalization results [43].

### Phenotype scanning

Given the limited number of SNPs included in MR, which restricts the ability to conduct sensitivity analyses for horizontal pleiotropy, we performed phenotype scanning to assess the associations of these SNPs with other traits. Phenotype scanning was conducted through GWAS ATLAS (https://atlas.ctglab.nl/) and GWAS Catalog (https://www.ebi.ac.uk/gwas/home) by manually searching each SNP across all previously reported GWAS datasets [44, 45]. An SNP was classified as pleiotropic if it met all the following criteria: (i) the association was identified in European ancestry populations; (ii) it reached genome-wide significance (*P* < 5 × 10^− 8^); (iii) it was associated with known risk factors for AD.

## Results

### Twenty-four candidate plasma proteins were identified in the discovery stage

**Fig. 2:**
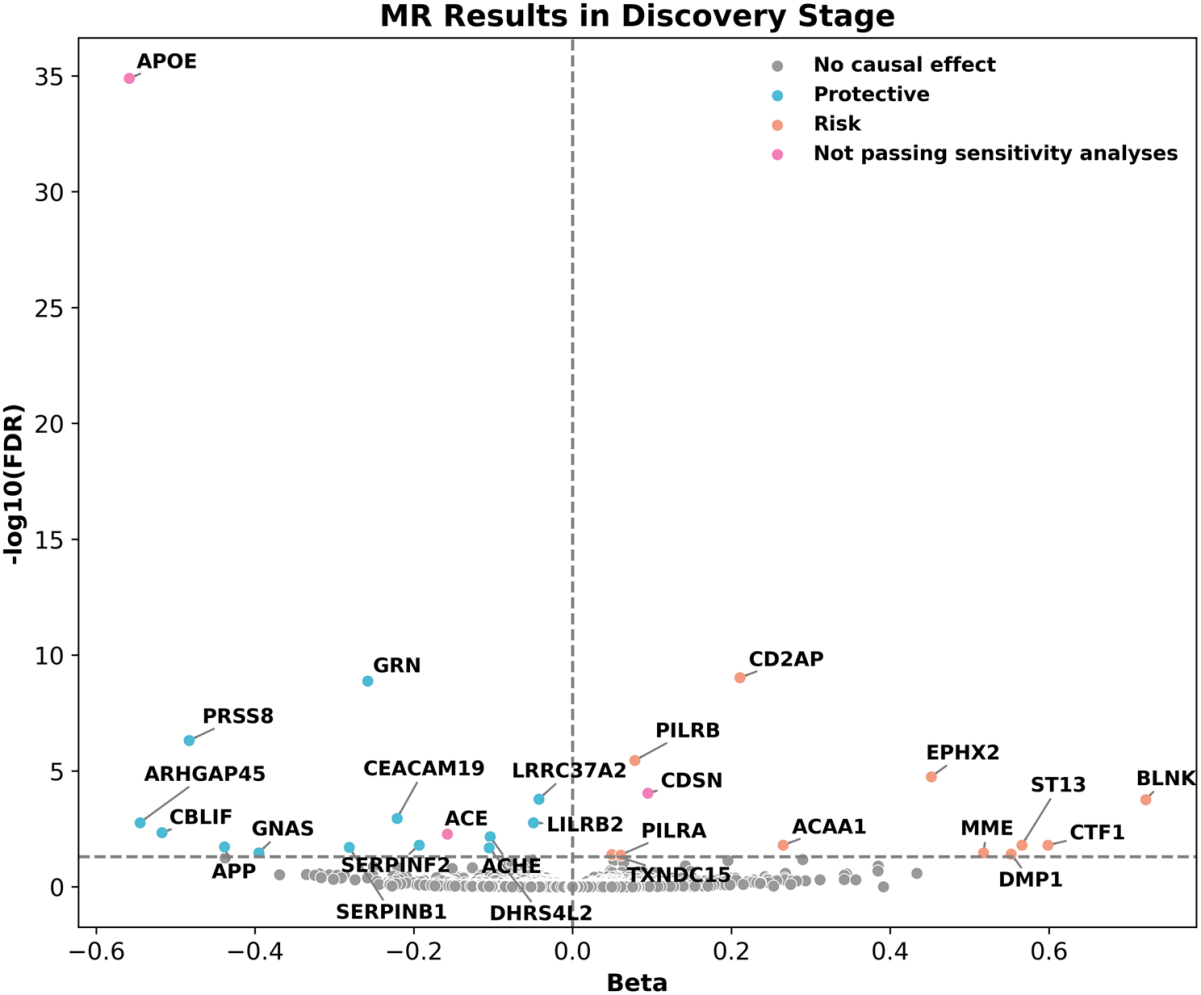
Volcano plot of plasma proteome-wide MR results in the discovery stage. MR analyses of plasma proteins on AD risk after correction with multiple testing in the discovery stage are highlighted in color. The beta from the Wald ratio or IVW method represents the causal estimate. Color differences represent different MR analysis results. Grey dots, no causal effect of proteins on AD; Blue dots, protective effect of proteins on AD; Orange dots, risk effect of proteins on AD; Pink dots, MR associations excluded by sensitivity analyses

According to Sun et al. [18], 1,916 of the 2,922 plasma proteins had at least one genetic association on the autosomes in *cis*-pQTLs at *P* < 1.7 × 10^− 11^. A total of 4,596 SNPs for 1,802 proteins remained after screening and harmonization. The SNPs for each protein were used as IVs for primary MR analyses evaluated by three methods, IVW, MR-Egger, and weighted median. As shown in Fig. 2, 27 genetically predicted plasma protein level changes had significant causal effects on AD after multiple testing corrections with FDR (*q* < 0.05; Additional File 1: Table S2). Three proteins were excluded from the subsequent analyses due to high heterogeneity, horizontal pleiotropy, or false causal direction after five additional tests in the sensitivity analyses. In detail, Cochran’s Q test results (*P* < 0.05) indicated the heterogeneity for apolipoprotein E (APOE) and angiotensin-converting enzyme (ACE) between the analyzed SNPs (Additional File 1: Table S2). The MR-Egger intercept p-value (*P* < 0.05) for ACE supported the horizontal pleiotropy conclusion (Additional File 1: Table S2). The MR-Steiger directionality test for corneodesmosin (CDSN) suggested a false causal direction (Additional File 1: Table S2). A true causal direction from proteins to AD was confirmed for the remaining 24 plasma proteins with MR-Steiger and Steiger filtering tests. We also conducted reverse MR analysis to test whether AS has causal effect on those 24 proteins with AD GWAS as exposure and pQTLs as an outcome. Of the 24 proteins, only 10 proteins were performed reverse MR, as no SNPs were left for 14 proteins after harmonization. For the 10 proteins tested, we did not see any evidence that AD has causal effects on the protein level change. See details in Additional File 1: Table S3.

**Fig. 3:**
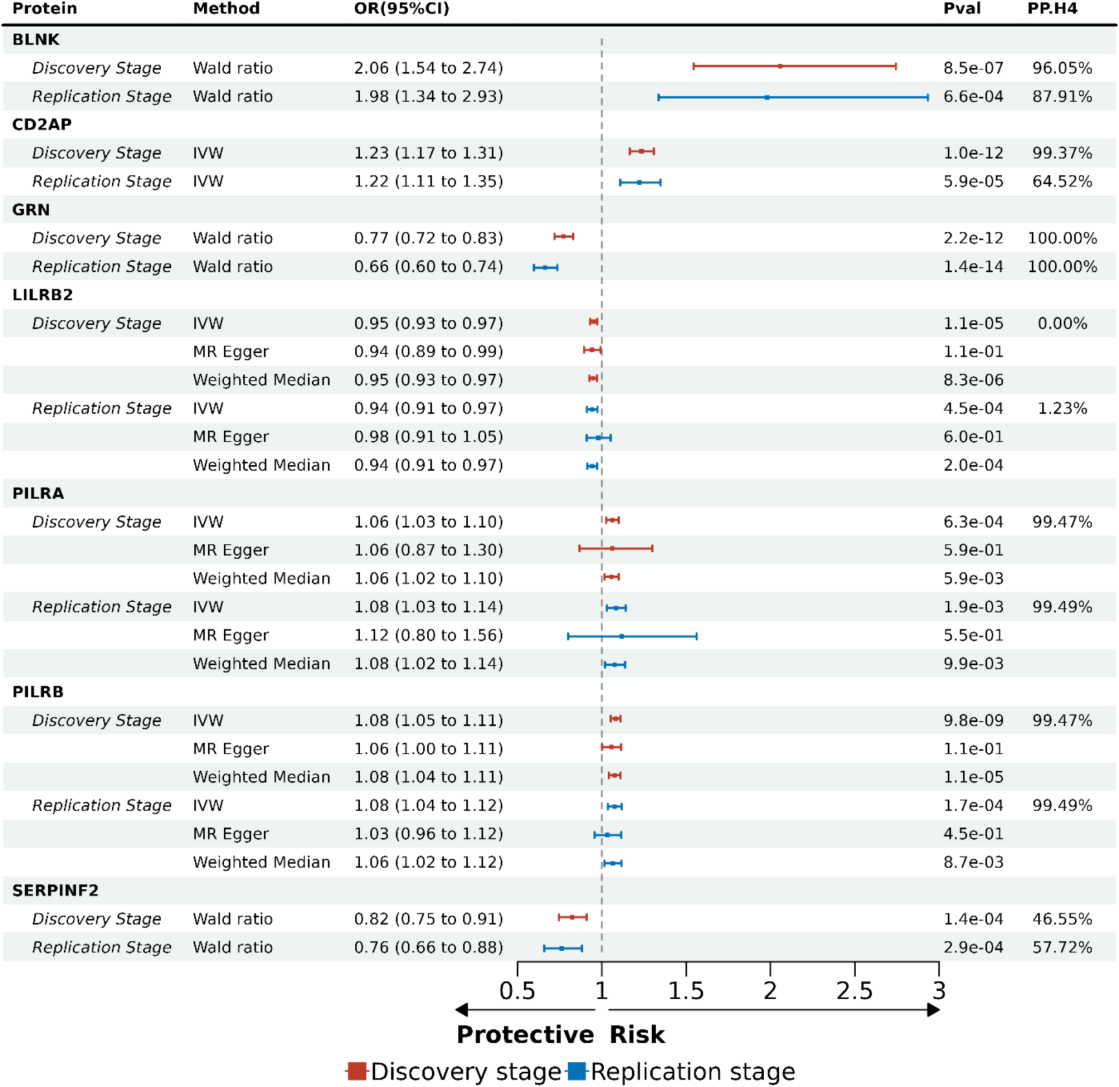
Summary of plasma proteome-wide MR and Bayesian colocalization analysis for seven validated causal proteins. The MR estimates and P values evaluated by Wald ratio, IVW, and weighted median methods for seven validated proteins, assessed in both the discovery and replication stages, are summarized in the table, as well as the posterior probability of hypothesis 4 of Bayesian colocalization analysis. The ORs with 95%CI for a one standard deviation change of genetically predicted protein levels on AD risk are presented, along with corresponding forest plots. In the forest plot, the length of the horizontal line represents the CI and the box at the center of each horizontal line represents the OR. Color differences represent different stages. Red, discovery stage; Blue, replication stage. IVW, inverse-variance weighted; OR, odds ratio; CI, confidential interval; Pval, P value; PP.H4, posterior probability of hypothesis 4

Of identified 24 plasma proteins, 15 proteins were estimated via the Wald ratio with only one SNP, 4 proteins were determined by IVW only with two SNPs, and 5 proteins were determined by IVW with more than two SNPs along with MR Egger and Weighted median (Additional File 1: Table S2). The estimates from the three methods showed consistent causal directions for the 5 plasma proteins, including paired immunoglobulin-like type 2 receptor alpha (PILRA), paired immunoglobulin-like type 2 receptor beta (PILRB), thioredoxin domain-containing protein 15 (TXNDC15), leukocyte immunoglobulin-like receptor subfamily B member 2 (LILRB2), and leucine-rich repeat-containing protein 37A2 (LRRC37A2). This consistency indicates that the MR results for the five proteins were robust and less likely to be influenced by pleiotropy of methodological artifacts. The scatter plots, forest plots, leave-one-out plots, and funnel plots of these five proteins are summarized in Additional File 2: Fig. S1. The causal estimates were reported as odds ratio (OR) with 95% confidential interval (CI) for one standard deviation (SD) change of genetically predicted plasma protein level on AD risk, as shown in Fig. 3. For instance, one SD increase of CD2-associated protein (CD2AP) (OR = 1.23, 95% CI: 1.17–1.31, *P* = 1.0 × 10^− 12^) in plasma was associated with 23% higher risk for AD, and one SD increase of progranulin (GRN) (OR = 0.77, 95% CI: 0.72–0.83, *P* = 2.2 × 10^− 12^) in plasma was associated with 23% lower risk for AD.

In summary, 24 plasma proteins were causally associated with AD in the discovery stage. The genetically predicted increase in the levels of 11 plasma proteins were associated with higher risk of AD, with the top five risk-associated proteins including B-cell linker protein (BLNK) (OR = 2.06, 95% CI: 1.54–2.74, *P* = 8.5 × 10^− 7^), cardiotrophin-1 (CTF1) (OR = 1.82, 95% CI: 1.33–2.48, *P* = 1.6 × 10^− 4^), Hsc70-interacting protein (ST13) (OR = 1.76, 95% CI: 1.31–2.36, *P* = 1.7 × 10^− 4^), dentin matrix acidic phosphoprotein 1 (DMP1) (OR = 1.74, 95% CI: 1.27–2.37, *P* = 5.3 × 10^− 4^), neprilysin (MME) (OR = 1.68, 95% CI: 1.26–2.24, *P* = 4.5 × 10^− 4^) (Additional File 1: Table S2). The genetically predicted increase in the levels of 13 plasma proteins was associated with reduced risk of AD, with the top five protective proteins including Rho GTPase-activating protein 45 (ARHGAP45) (OR = 0.58, 95% CI: 0.46–0.74, *P* = 1.0 × 10^− 5^), cobalamin binding intrinsic factor (CBLIF) (OR = 0.60, 95% CI: 0.47–0.76, *P* = 3.3 × 10^− 5^), prostasin (PRSS8) (OR = 0.62, 95% CI: 0.53–0.72, *P* = 1.1 × 10^− 9^), amyloid-beta precursor protein (APP) (OR = 0.64, 95% CI: 0.51–0.81, *P* = 2.1 × 10^− 4^), and neuroendocrine secretory protein 55 (GNAS) (OR = 0.67, 95% CI: 0.54–0.84, *P* = 4.6 × 10^− 4^) (Additional File 1: TableS2).

### Seven AD-associated plasma proteins were validated in the replication stage

In the replication stage, we sought to validate the 24 candidate plasma proteins identified in the discovery stage using independent AD GWAS data, FinnGen_R11, as the outcome. The MR analysis was performed using the same pipeline as in the discovery stage. Seven genetically predicted plasma protein levels were replicated as causally associated with AD at *P* < 2.08 × 10^− 3^ (0.05/24 with Bonferroni correction, Additional File 1: Table S4). As shown in Fig. 3, the casual directions of all seven validated proteins were consistent with those from the discovery stage. Further MR-Steiger test confirmed the true causal direction. The genetically predicted plasma protein level increases of BLNK (OR = 1.98, 95% CI: 1.34–2.93, *P* = 6.6 × 10^− 4^), CD2AP (OR = 1.22, 95% CI: 1.11–1.35, *P* = 5.9 × 10^− 5^), PILRA (OR = 1.08, 95% CI: 1.03–1.14, *P* = 1.9 × 10^− 3^), and PILRB (OR = 1.08, 95% CI: 1.04–1.12, *P* = 1.7 × 10^− 4^) were associated with higher AD risk. While the genetically predicted increases of GRN (OR = 0.66, 95% CI: 0.60–0.74, *P* = 1.4 × 10^− 14^), alpha-2-antiplasmin (SERPINF2) (OR = 0.75, 95% CI: 0.65–0.88, *P* = 3.0 × 10^− 4^) and LILRB2 (OR = 0.94, 95% CI: 0.91–0.97, *P* = 4.9 × 10^− 4^) protein in plasma showed protective associations with AD. No heterogeneity or horizontal pleiotropy was detected in the sensitivity analyses for protein CD2AP, LILRB2, PILRA, and PILRB. The sensitivity analyses of horizontal pleiotropy for CD2AP were not available because only two SNPs were left after harmonization. The scatter plots, forest plots, leave-one-out plots, and funnel plots of other three validated MR associations are summarized in Additional File 2: Fig. S2. For protein BLNK and GRN, the sensitivity analyses were not available because only one SNP was left after harmonization. Six plasma proteins demonstrated consistent causal directions in both discovery and replication stages, although their causal effects on AD were not statistically significant after Bonferroni correction (2.08 × 10^− 3^ < *P* < 0.05) in the replication stage. Among these six proteins, bifunctional epoxide hydrolase 2 (EPHX2) showed risk effect on AD, while acetylcholinesterase (ACHE), APP, carcinoembryonic antigen-related cell adhesion molecule 19 (CEACAM19), GNAS, and PRSS8 showed protective effects against AD.

### Five plasma proteins were confirmed by colocalization

Bayesian colocalization analysis was conducted for each of the 7 validated plasma proteins across the discovery and replication stages. Among the seven validated plasma proteins, four demonstrated strong colocalization with AD in both the discovery and replication stages, including BLNK (discovery stage PP.H_4_ = 96.05%, replication stage PP.H_4_ = 87.91%), GRN (discovery stage PP.H_4_ = 100%, replication stage PP.H_4_ = 100%), PILRA (discovery stage PP.H_4_ = 99.47%, replication stage PP.H_4_ = 99.49%), and PILRB (discovery stage PP.H_4_ = 99.47%, replication stage PP.H_4_ = 99.49%) (Fig. 3; Additional File 1: Table S5). Protein CD2AP (discovery stage PP.H_4_ = 99.37%, replication stage PP.H_4_ = 64.52%) showed strong colocalization only in the discovery stage. These high PP.H4 values indicate shared causal variants between these plasma proteins and AD. Plasma proteins showing strong colocalization in either both discovery stage and replication stages or in one stage were confirmed as putative proteins. Colocalization results revealed evidence of indirect horizontal pleiotropy in the association between LILRB2 and AD (PP.H_3_ = 99.85%). This indicates that the observed MR association was likely driven by confounding variants in linkage disequilibrium (LD), with distinct causal variants independently affecting LILRB2 protein levels and AD risk. Both PP.H_3_ and PP.H_4_ of protein SERPINF2 were less than 80%, indicating the absence of sufficient statistical power to distinguish whether the MR association between SERPINF2 and AD was driven by a shared causal variant or a confounding variant in LD. Overall, five plasma proteins—BLNK, CD2AP, GRN, PILRA, and PILRB—were confirmed by colocalization. We considered those five plasma proteins as putative proteins/candidates. The regional association plots for colocalization results of the five plasma proteins are summarized in Additional File 2: Fig. S3.

### Phenotype scanning for putative causal proteins

Among five putative proteins, sensitivity analyses for accessing horizontal pleiotropy were not available for BLNK, CD2AP, and GRN due to limited number of SNPs included in MR. In phenotype scanning (Additional File 1: Table S6), BLNK-related SNP (rs55769428) was not significantly associated with any traits at *P* < 5 × 10^− 8^. For CD2AP, one of its related SNP (rs5516297) was not significantly associated with any traits, while another SNP (rs13212790) was observed to be associated with several traits, including AD, standing height traits and blood cell traits, such as red cell distribution width, mean platelet volume, platelet distribution width and so on. The associations between SNP (rs13212790) and AD were derived from proxy and clinically diagnosed AD traits, which were likely part of AD GWAS used as MR outcome in our discovery stage [46]. GRN-related SNP (rs5848) was found to be associated with multiple traits, including AD, blood cell traits, long sleep, granulins levels, GRN levels, completement C1q tumor necrosis factor-related protein 1 (C1QTNF1) levels, and memory. The associations between SNP (rs5848) and AD were derived from the same AD GWAS used in our discovery stage. Although both CD2AP-related SNP (rs13212790) and GRN-related SNP (rs5848) were associated with AD, Steiger filtering indicated that these SNPs are unlikely to have direct effects on AD.

### Associations of 5 protein-coding genes with AD

**Fig. 4:**
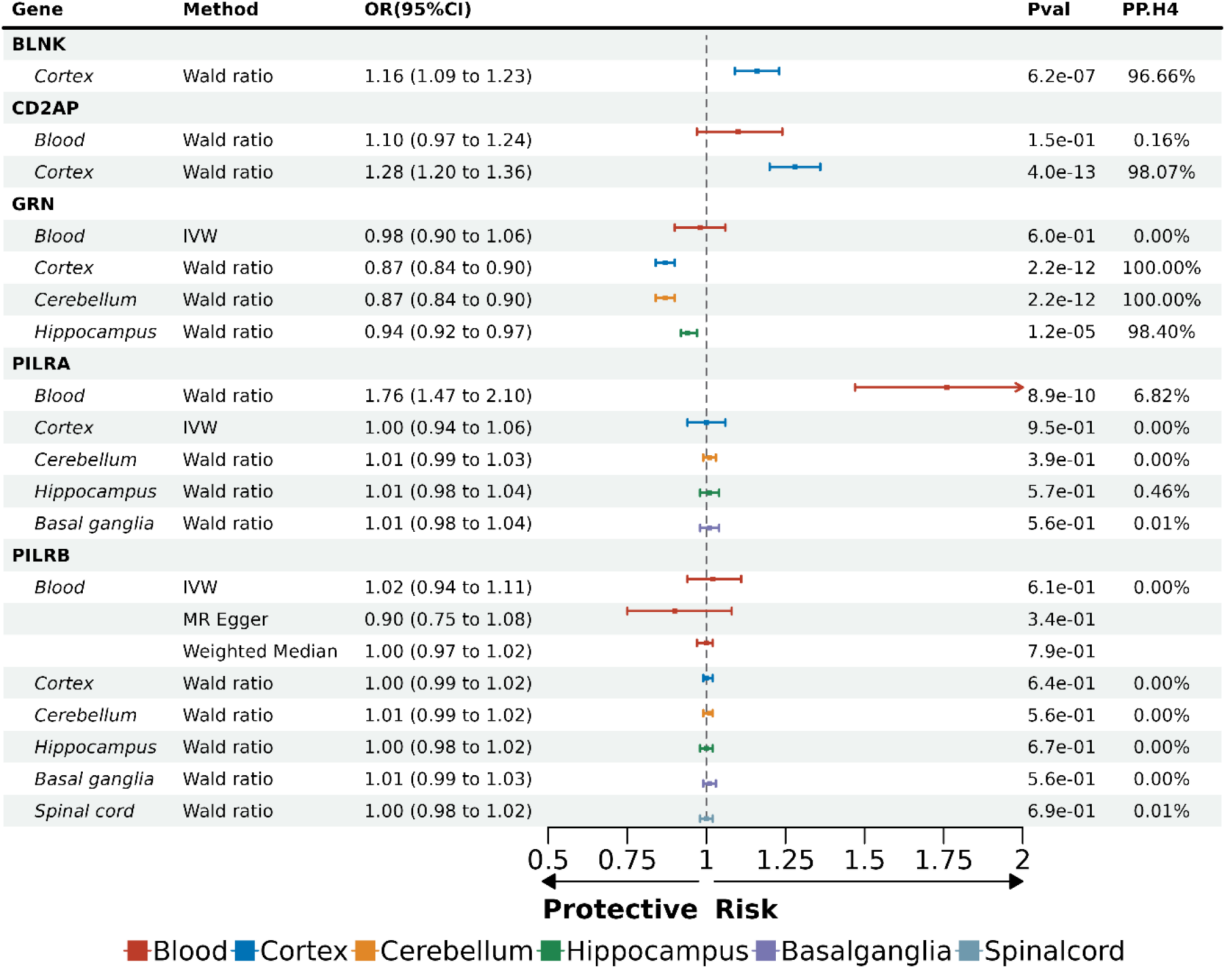
Summary of transcriptome-wide MR and Bayesian colocalization analysis results of five causal protein-coding genes. The MR estimates and P values evaluated by Wald ratio, IVW, and weighted median methods for five putative causal protein-coding genes across different tissues are summarized in the table, as well as the posterior probability of hypothesis 4 of Bayesian colocalization analysis. The ORs with 95%CI for a one standard deviation change of genetically predicted gene expression on AD risk are presented, along with corresponding forest plots. In the forest plot, the length of the horizontal line represents the CI and the box at the center of each horizontal line represents the OR. Color differences represent different stages. Color differences represent different tissue types. Red, blood; Blue, cortex; Orange, cerebellum; Green, hippocampus; Purple, basal ganglia; Light blue, spinal cord. IVW, inverse-variance weighted; OR, odds ratio; CI, confidential interval; Pval, P value; PP.H4, the posterior probability of hypothesis 4

To further explore the causal associations between the expression of corresponding protein-coding genes and AD, transcriptome-wide MR analysis was performed between *cis*-eQTLs of five putative protein-coding genes and AD, followed by colocalization analysis. Five coding genes were mapped for the five putative plasma proteins. The blood and brain *cis*-eQTLs of these five coding genes used as exposure in MR were collected from eQTLGen Consortium and MetaBrain, respectively. The AD GWAS dataset used as outcome in MR was the discovery data of Bellenguez et al.’s study [9]. In blood, only *PILRA* (OR = 1.76, 95% CI: 1.47–2.10, *P* = 8.9 × 10^− 10^) showed a consistent association with higher AD risk in the eQTLGen dataset, while the colocalization results indicated that the association was due to horizontal pleiotropy. For five brain regions, *BLNK* (OR = 1.16, 95% CI: 1.09–1.23, *P* = 6.2 × 10^− 7^) and *CD2AP* (OR = 1.28, 95% CI: 1.20–1.36, *P* = 4.0 × 10^− 13^) presented consistent associations with higher AD risk in the cortex, and *GRN* demonstrated consistent protective associations with AD in the cerebellum (OR = 0.87, 95% CI: 0.84–0.90, *P* = 2.2 × 10^− 12^), cortex (OR = 0.87, 95% CI: 0.84–0.90, *P* = 2.2 × 10^− 12^) and hippocampus (OR = 0.94, 95% CI: 0.92–0.97, *P* = 1.2 × 10^− 5^): these associations were supported by strong colocalization evidence (Fig. 4; Additional File 1: Tables S7, S8). Confirmed by colocalization analysis, the consistent causality between transcriptome-wide MR in brain regions and proteome-wide MR in plasma for *BLNK*, *CD2AP* and *GRN* strengthen the robustness of our findings, suggesting their potential as systemic biomarker and therapeutic target. No evidence of causal associations between expression levels of *BLNK*, *CD2AP* and *GRN* in blood and AD was found. Similarly, *PILRB* expression showed no causal effect on AD either in blood or brain. Transcriptome-wide MR analysis could not be conducted for several genes in blood and brain tissues because no SNP remained after harmonization. The absence of overlapping genetic instruments between *cis*-eQTLs (exposure) and AD (outcome) suggests that the currently available data lack sufficient statistical power to investigate their casual associations. The regional association plots for colocalization results of putative protein-coding genes are summarized in Additional File 2: Fig. S3.

### Replication of causal plasma proteins in African ancestry

To identify plasma proteins causally associated with AD across different ethnic groups, we conducted proteome-wide MR utilizing *cis*-pQTLs and AD GWAS data derived from populations of African ancestry. In date from those of African ancestry, 639 plasma autosomal *cis*-pQTLs have been identified at *P* < 1.7 × 10^− 11^ from 931 individuals in Sun et al.’s study [18]. After harmonization, a total of 607 SNPs for 560 plasma proteins were used as IVs for primary MR analysis. No causal effect was found from any plasma proteins at *q* < 0.05 in date from individuals of African ancestry. Among five putative plasma proteins identified in European ancestry, only two proteins, PILRA and PILRB, were among the 560 proteins (Additional File 1: Table S9). In our study, PILRA and PILRB were causally associated with AD only in European ancestry populations, indicating the existence of population-specific protein risk factors for AD. Given that the sample size of African ancestry datasets is considerably smaller than that of European ancestry datasets, the absence of causal effects may also be attributed to the limited statistical power of the datasets used in our study. This underscores the critical need for large-scale genetic studies involving diverse populations to eliminate the statistical bias.

### Summary of the findings

The findings of this study are summarized in Fig. 1 and Additional File 1: Table S10. Five plasma proteins were considered putative candidates associated with AD through a comprehensive approach that combined proteome-wide MR, transcriptome-wide MR, Bayesian colocalization analysis and phenotype scanning. Based on evidence from the discovery stage, replication stage, and replication at the transcriptional level, we stratified the five causal proteins into two tiers and compared them with previous MR studies. Proteins that demonstrated consistent significant causal associations across all analyses, including in the discovery stage, replication stage, and transcriptional-level replication, were classified as Tier 1. Proteins that showed significant causal associations in proteome-wide MR analyses in both the discovery and replication stages and either (i) were validated by Bayesian colocalization analysis in at least one stage or (ii) demonstrated causal effects on AD at the transcriptional level, were classified as Tier 2. Tier 1 included 2 proteins, BLNK and GRN, that passed proteome-wide MR and colocalization analysis in the discovery and replication stages, as well as brain transcriptome-wide MR analyses confirmed by colocalization. Tier 2 included 3 proteins, of which PILRA and PILRB passed all analyses in the discovery and replication stages. *PILRA* demonstrated a significant association in blood transcriptome-wide MR analysis; however, no evidence of colocalization was observed at the transcriptome-wide level. *PILRB* did not show any transcriptional association with AD in any brain regions or blood. CD2AP remained a putative causal protein for AD, supported by significant associations in both plasma proteome-wide and brain transcriptome-wide MR analyses, despite lacking strong colocalization with AD in the replication stage. Among the five putative plasma proteins, GRN and PILRA had been previously implicated in MR studies, whereas BLNK, CD2AP, and PILRB emerged as novel causal candidates.

## Discussion

In this study, our comprehensive pipeline included a series of stringent approaches with primary MR analysis, sensitivity analyses, reverse MR analysis, replicative validation, Bayesian colocalization analysis, and phenotype scanning. Our study identified five putative plasma proteins—BLNK, CD2AP, GRN, PILRA, and PILRB—that were causally associated with AD predisposition in European-ancestry populations, highlighting their potential as diagnostic biomarkers or therapeutic drug targets. We are the first to identify a causal relationship between the three proteins—BLNK, CD2AP, and PILRB—and AD predisposition. The two proteins GRN and PILRA were identified as being associated with AD in earlier MR studies [47–51]. The increased genetically predicted levels of plasma proteins BLNK, CD2AP, PILRA, and PILRB were associated with higher AD risk, while the increased level of GRN was associated with lower AD risk. Of the five proteins, BLNK and GRN were associated with AD in both plasma proteome-wide and brain transcriptome-wide MR analyses. On the other hand, PILRB is associated with AD risk only in plasma proteome-wide MR analyses. PILRA exhibited a consistent causal effect on AD in plasma proteome-wide and blood transcriptome-wide MR analyses, while its transcriptional MR association was not supported by colocalization analysis. CD2AP showed significant causal associations in both plasma proteome-wide MR from the discovery stage and brain transcriptome-wide MR analyses, while its plasma proteome-wide MR result from the replication stage lacked colocalization evidence. In the African ancestry group, no causal associations were found between plasma proteins and AD. In this study, we prioritized five plasma proteins as potential diagnostic biomarkers or drug targets for AD in European ancestry populations, categorizing them into two tiers based on their relevance and potential impact.

Examining the MR results of five putative proteins evaluated at both the transcriptional and protein levels, we found that they did not show significant causal associations with AD in blood *cis*-eQTLs as observed in plasma *cis*-pQTLs. The discordance between protein levels and their corresponding mRNA levels, as frequently observed in large cohorts, may stem from additional regulatory processes beyond transcription that influence protein expression, including variations in translation rates, modulation of protein stability and half-life, synthesis delays, and transport mechanisms [52, 53]. Moreover, protein levels in the plasma may be affected by brain-derived factors due to blood-brain barrier dysfunction or specific transport mechanisms, such as the bidirectional crossing of the blood-brain barrier by tau proteins [54, 55]. While the five putative proteins did not show significant associations in blood transcriptome-wide MR, they remain promising candidates for further investigation into their potential roles in AD. Two of the five identified proteins, CD2AP and GRN, were found to be associated with other traits via phenotype scanning, however, these associations could not fully explain their relationships with AD. For example, CD2AP-related SNP (rs13212790) was associated with standing height traits, which is not likely bias the CD2AP-AD relationship [44]. C1QTNF1 was found to be *trans*-associated with GRN-related SNP (rs5848) [56, 57], and showed no significant causal relationship with AD in our discovery stage, suggesting that it is less likely to bias the GRN-AD association. CD2AP (rs13212790) and GRN (rs5848) were both linked to blood cell traits, with rs5848 primarily associated with platelet traits and rs13212790 also associated with red cell and reticulocyte traits [58]. However, previous MR studies have showed no causal relationship between those blood cell traits and AD predisposition [58, 59], indicating that blood cell traits are unlikely to bias the observed associations between identified proteins and AD. Granulin, the precursor of progranulin, was previously reported to be casually associated with platelet count in an MR study, a finding further validated by gene knockdown experiments in cells [60]. Progranulin regulates lysosomal function, playing a key role in inflammation and immunomodulation in the pathogenesis of cardiovascular diseases (CVD) [61]. The evidence suggests that GRN might serve as a biological link between AD and CVD. It was also found that rs5848 was associated with cognitive scores for memory, which is the commonly used to assess AD progression [62]. Phenotype scanning also revealed the association between rs5848 and long sleep duration [63]. Previous study reported that AD risk was higher in the long sleep duration group without MR causal relationship, while another MR study showed that AD risk was negatively associated with long sleep duration [64, 65]. Due to inconsistent findings, the role of long sleep duration in the GRN-AD association remains uncertain and warrants further investigation.

Our study shows that higher genetically predicted levels of the BLNK protein in plasma and higher expression of the *BLNK* gene in the cortex are correlated to an increased risk of AD. BLNK is an adaptor protein of the B-cell receptor signaling pathway and has been identified as an independent risk factor for colorectal cancer recurrence as well as a positive regulator of Met signaling in non-small cell lung cancer [66, 67]. It has been observed that the tumor suppressor protein p53 plays a role in the upregulation of *BLNK* with the activation of immune cells [68]. Notably, the microglia receptor TREM2 is a direct p53 target gene, suggesting that *BLNK* is involved in the signaling pathway initiated by *TREM2* [69]. Studies have shown that the *BLNK* gene is predominantly expressed in microglia, and its upregulation in response to Aβ exposure suggests a role in microglial responses to Aβ pathology [9, 70]. Monitoring or targeting BLNK has the potential to understand the pathological progression of AD, but further studies are required.

We found that higher levels of the CD2AP protein in plasma and higher expression of the *CD2AP* gene in the cortex increase the risk for AD. CD2AP is a multifunctional adaptor protein crucial for maintaining neuronal homeostasis, synaptic function, neurite structure, blood-brain barrier integrity, and calcium physiology [71–73]. Genetic variants in the *CD2AP* locus are associated with increased CSF tau levels in mild cognitive impairment, decreased cognitive function in middle-aged individuals with a family history of AD, and increased risk of AD, all-cause dementia, and vascular dementia [74–76]. Previous study reported that CD2AP enhances the degradation of amyloid precursor protein by accelerating its transition from early to late endosomes in neurons in vitro [77]. However, another study reported that CD2AP loss of function did not exacerbate Aβ deposition or accumulation in an AD mouse model [78]. Xue et al. found that neuronal CD2AP deficiency exacerbated tau phosphorylation, synaptic injury, and cognitive impairment in an AD mouse model [79]. In contrast, CD2AP neuronal expression was positively associated with Braak neurofibrillary tangle stage in AD patients [80]. These discrepant findings highlight the complex role of neuronal CD2AP expression in Aβ metabolism and tau pathology, suggesting CD2AP as a promising therapeutic target for AD. Further longitudinal studies of CD2AP levels in plasma are required to evaluate its potential as a biomarker for AD.

Progranulin (encoded by the *GRN* gene), a glycoprotein mainly secreted by activated microglial cells in the brain, binds to Sortilin (encoded by the *SORT1* gene) expressed on neuronal cell surfaces, leading to rapid endocytosis and delivery to the lysosome for degradation [81–83]. Mutations in the *GRN* gene are commonly linked to familial frontotemporal dementia [84]. Progranulin has been negatively associated with the risk of Parkinson’s disease [85]. In prior MR studies, higher progranulin levels in both CSF and blood were associated with a protective effect in AD [47–49]. Progranulin is the sole protective protein against AD we identified through MR, consistent with previous studies. In our study, the rs5848 T allele is associated with a 22% reduction of progranulin in plasma and is linked to a 23% increased risk of AD through MR analysis using the Wald ratio method. Higher *GRN* expression in the brain was associated with a lower risk of AD in our transcriptome-wide MR analyses. Additionally, rs5848 has been reported to be associated with hippocampal sclerosis and TDP-43 deposits [86]. Progranulin is considered a promising therapeutic target for neurodegenerative diseases, including AD [87]. Currently, an anti-sortilin monoclonal antibody (GSK4527226), increasing progranulin levels in plasma and brain via inhibiting its binding with Sortilin, is entering a Phase 2 clinical trial to assess safety and efficacy in patients with early AD [88].

PILRA is an inhibitory receptor expressed on the surface of immune cells including microglia [89]. Several studies have shown that a common missense variant of *PILRA* (G78R, rs1859788) is protective against AD by reducing its binding to several ligands in microglia. Because of this, *PILRA* might decrease microglial infection by the herpes simplex virus 1, a virus linked to AD risk [89–91]. Debette et al. reported that the higher genetically determined levels of two soluble isoforms of PILRA (deltaTM and M14) in CSF and plasma are associated with a lower risk of AD [51]. Plasma PILRA was found to be negatively associated with AD in another MR study with the same proteome data as in Debette et al.’s study [50]. *Cis*-regulated levels of PILRA in CSF were negatively associated with AD in a study by Cruchaga et al. [47]. In contrast, our study showed that a higher genetically predicted level of PILRA in plasma is associated with a higher risk of AD, consistent with previous studies. It is not clear why different effects of PILRA on AD have been reported. We note that the proteomic levels of the three studies with negative associations were measured by aptamer-based SomaScan assay, while the proteomic levels of our study were measured by the antibody-based Olink assay. Pietzner et al. reported a discordant association direction between the missense variant rs1859788 and PILRA levels measured by Olink and SomaScan assays [92]. The discrepancy in the results is likely due to differences between the platforms used for protein measurement, as the two assays likely capture distinct aspects of protein chemistry. A recent study found that the Olink platform had higher protein target specificity and robust phenotypic associations and that the SomaScan platform exhibited greater measurement precision and breadth [93]. Another study suggested that each protein might have different effects across different tissues [94]. Hence, multi-platform proteomics across CSF and plasma is a promising strategy for further clarification of PILRA function in AD. Park et al. found that the *PILRA* gene is significantly upregulated in blood transcriptome data from patients with AD compared to normal controls [95]. In addition, Smith et al. reported that the *PILRA* gene is upregulated in microglia isolated from neuropathologically defined AD brains and is correlated with Aβ and p-tau expression [96]. According to Monroe et al., a high-affinity PILRA antagonist antibody phenocopying PILRA loss-of-function has been identified as a therapeutic approach for AD [97]. Despite the conflicting causal effects reported in different studies, we still consider PILRA as a promising druggable target for AD.

PILRB is expressed in inflammatory macrophages, natural killer cells, dendritic cells, and microglial cells, all of which contribute to the production of inflammatory factors during infection [98, 99]. Our MR studies are the first to report that increased PILRB abundance is associated with a higher risk of AD. Since PILRB and PILRA belong to the same family, further research is needed to elucidate their respective roles in AD pathology.

Our findings are corroborated by existing literature, demonstrating that MR serves as a promising proxy approach for prioritizing potential diagnostic biomarkers and drug targets for subsequent validation. The five putative proteins are mainly related to microglial activities and lysosomal functions, supporting a pivotal role of microglia and lysosomes in AD. Notably, higher plasma APP levels were associated with a lower risk for AD risk in our discovery stage. Although the association showed a similar trend in the replication stage, it did not reach statistical significance. The result seems paradoxical given that over dosage of *APP* gene in Down syndrome is linked to early-onset AD [100, 101]. This discrepancy emphasizes the need for further studies on tissue-specific expression, isoform-specific effects and post-translational modification patterns of APP to better understand its complex role in AD. Other studies have reported that PRSS8 was associated with an increased risk of AD in CSF but a reduced risk in plasma [47, 57]. Su et al. prioritized druggable gene *EPHX2* as a potential therapeutic target for AD through transcriptome-wide and phenome-wide MR analyses [102]. However, PRSS8 and EPHX2 were excluded in the replication stage as they failed to meet statistical significance after multiple testing correction. Further studies on these proteins are needed to clarify their true association with AD.

Our study has several strengths. First, genetic instruments identified in a large-scale proteogenomic study were robust and powerful allowing us to explore the causal effect of plasma protein abundance on AD liability. By mimicking biomarker fluctuations and drug-target interactions observed in clinical trials, our comprehensive MR analyses provided an efficient and cost-effective approach to evaluate causality for the development of diagnosis biomarkers and drug targets for AD. Second, we systematically investigated and validated causal relationships between plasma protein and AD risk through two-sample MR analyses, leveraging large-scale *cis*-pQTL data and two independent AD GWAS datasets. A series of rigorous sensitivity analyses, reverse MR analysis, and Bayesian colocalization analysis were conducted to ensure the robustness and reliability of identified causal associations. Third, the consistent results observed in both proteomics and transcriptional levels across blood and brain tissues further strengthen the robustness of the findings. Last, we explored the causal effect between plasma pQTLs and AD in African ancestry. The putative causal associations identified in European ancestry were not replicated in African ancestry, highlighting potential differences in AD pathology in different ethnic groups.

This study also has limitations. First, different protein measurement platforms may capture distinct isoforms, potentially leading to opposite causal directions as seen with PILRA in our MR analysis. Notably, evaluating causal associations between plasma protein levels and AD risk through genetically predicted changes from large populations may introduce inherent bias. Thus, validation of these findings requires measurements of plasma protein changes from individual-level data across multiple analytical platforms. Second, several MR associations were identified only when using the Wald ratio method due to a limited number of valid SNP IVs after harmonization, which did not allow further sensitivity analyses. Although colocalization analysis and phenotype scanning were conducted, not all potential horizontal pleiotropy or confounding factors could be eliminated. Third, not all of the pQTLs are available in African ancestry data, thus, it is impossible to fully compare our results across different ethnic groups. In addition, the sample size for African ancestry is much smaller compared to that for European ancestry, which may reduce the power of the analysis. Further validation of our findings would require functional assessment of these biomarkers or targets in either induced pluripotent stem cells or animal models of AD. Ultimately, therapeutic validation would entail implementing these observations in clinical trials.

Blood-based biomarkers have the potential to enhance patient recruitment and stratification for clinical trials and advance the development of diagnostics and precision medicine. Recently, Jiang et al. reported a blood-based 21-protein assay that captures changes in multiple biological pathways, accurately classifying AD and mild cognitive impairment across different ethnic populations [103]. Plasma biomarkers prioritized through MR, followed by further validation and mechanistic studies, have enormous potential for improving the diagnosis and advancing therapeutic development for AD. By using the expression levels of drug targets as proxies for drug exposure and employing genetic variants associated with the expression level of drug targets as IVs for MR analysis, the potential effects of drug target inhibitors on the risk of other diseases can be inferred [104, 105]. A large sample of proteomic data from diverse ethnic groups, measured across different platforms, is essential for identifying potential protein biomarkers or drug targets for AD.

In conclusion, five plasma proteins demonstrated causal effects for AD in European ancestry populations. Among these, BLNK and GRN were prioritized as Tier 1 causal proteins for AD, while CD2AP, PILRA, and PILRB were nominated as Tier 2 causal proteins for AD. Further studies with these five plasma proteins could provide more insights into AD pathology, potentially serving as blood-based biomarkers for disease diagnosis and therapeutic targets for the treatment.

## Electronic supplementary material

Below is the link to the electronic supplementary material.

Supplementary Material 1

Supplementary Material 2

## Abbreviations

AD: Alzheimer’s disease
MR: Mendelian randomization
cis-pQTL: cis protein quantitative trait loci
GWAS: Genome-wide association studies
cis-eQTL: cis gene expression quantitative trait loci
Aβ: Amyloid-beta
CSF: Cerebrospinal fluid
SNPs: Single nucleotide polymorphisms
Mb: Megabase
TSS: Transcription start site
UKB-PPP: UK Biobank Pharma Proteomics Project
IVs: Instrumental variables
IVW: Inverse-variance weighted
FDR: False discovery rate
PP.H3: Posterior probability of hypothesis 3
PP.H4: Posterior probability of hypothesis 4
OR: Odds ratio
CI: Confidential interval
SD: Standard deviation
LD: Linkage disequilibrium
mRNA: Messenger RNA
C1QTNF1: Completement C1q tumor necrosis factor-related protein 1
CVD: Cardiovascular diseases
APOE: (Apolipoprotein E)
CD2AP: (CD2-associated protein)
GRN: (Progranulin)
PRSS8: (Prostasin)
PILRB: (Paired immunoglobulin-like type 2 receptor beta)
EPHX2: (Bifunctional epoxide hydrolase 2)
CDSN: (Corneodesmosin)
LRRC37A2: (leucine-rich repeat-containing protein 37A2)
BLNK: (B-cell linker protein)
CEACAM19: (Carcinoembryonic antigen-related cell adhesion molecule 19)
ARHGAP45: (Rho GTPase-activating protein 45)
LILRB2: (Leukocyte immunoglobulin-like receptor subfamily B member 2)
CBLIF: (Cobalamin binding intrinsic factor)
ACE: (Angiotensin-converting enzyme)
ACHE: (Acetylcholinesterase)
SERPINF2: (Alpha-2-antiplasmin)
CTF1: (Cardiotrophin-1)
ACAA1: (3-ketoacyl-CoA thiolase, peroxisomal)
ST13: (Hsc70-interacting protein)
APP: (Amyloid-beta precursor protein)
SERPINB1: (Leukocyte elastase inhibitor)
DHRS4L2: (Dehydrogenase/reductase SDR family member 4-like 2)
GNAS: (Neuroendocrine secretory protein 55)
MME: (Neprilysin)
DMP1: (Dentin matrix acidic phosphoprotein 1)
TXNDC15: (Thioredoxin domain-containing protein 15)
PILRA: (Paired immunoglobulin-like type 2 receptor alpha)
