# Supplementary material for: Biomarker identification for Alzheimer’s disease through integration of comprehensive Mendelian randomization and proteomics data
Source: J Transl Med. 2025 Mar 6;23:278. doi: 10.1186/s12967-025-06317-5 (PMC11884171; doi:10.1186/s12967-025-06317-5)
Supplement: Supplementary file 2 — Supplementary Material 2 [file 12967_2025_6317_MOESM2_ESM.docx]

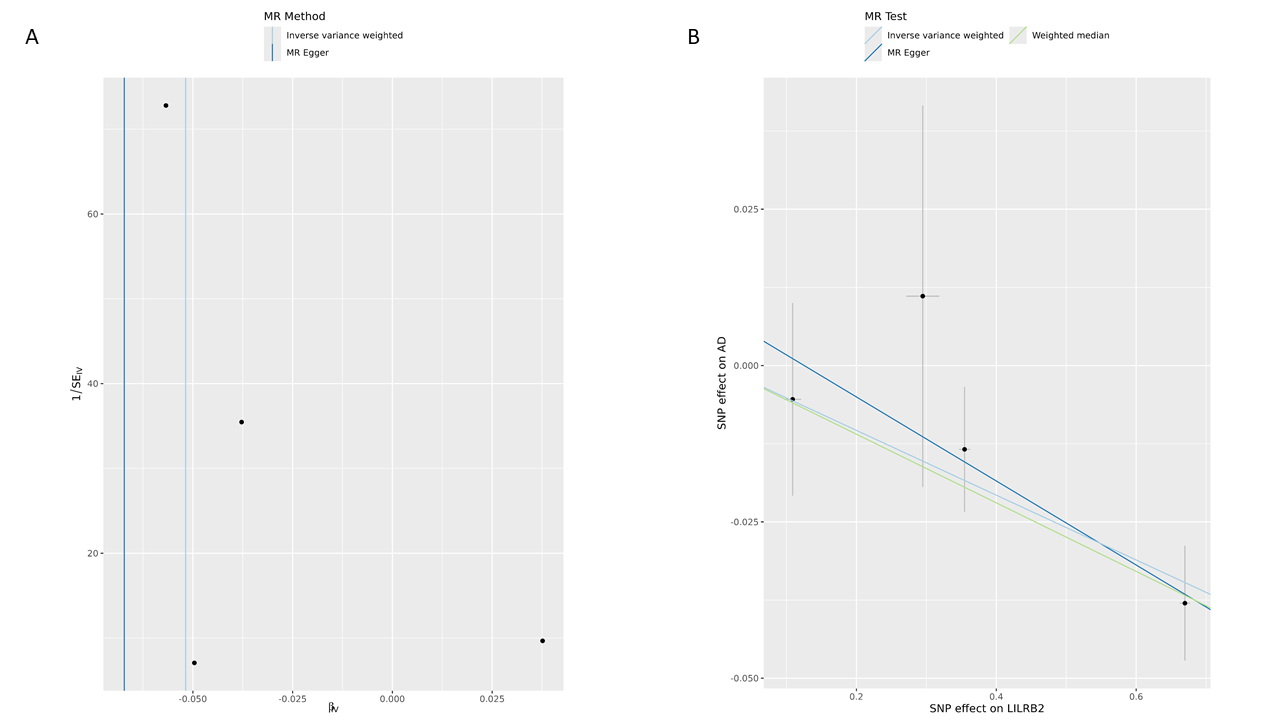


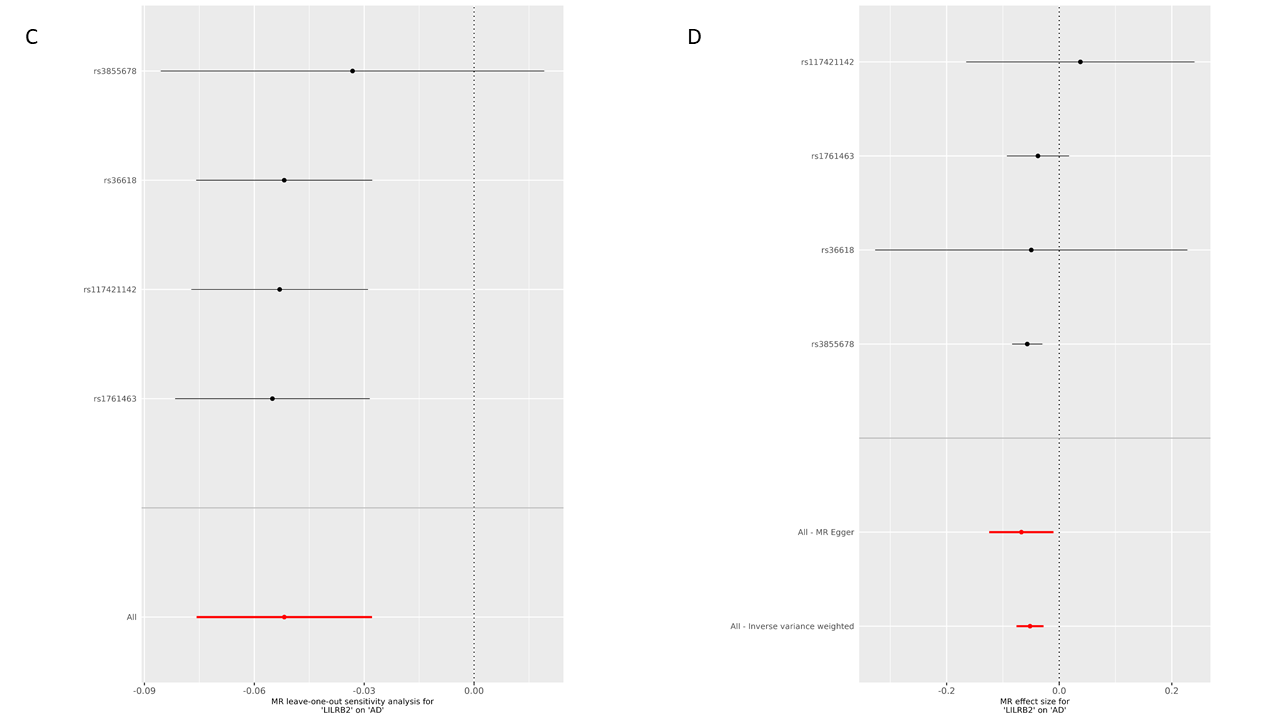


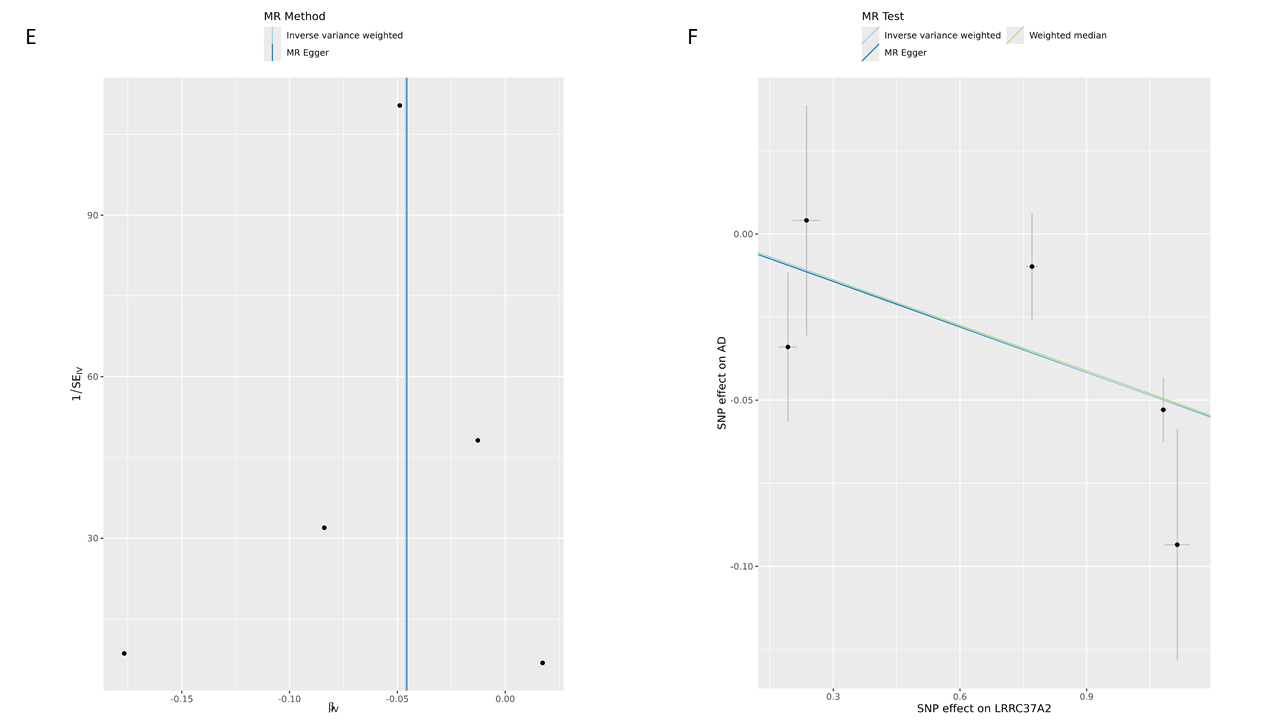


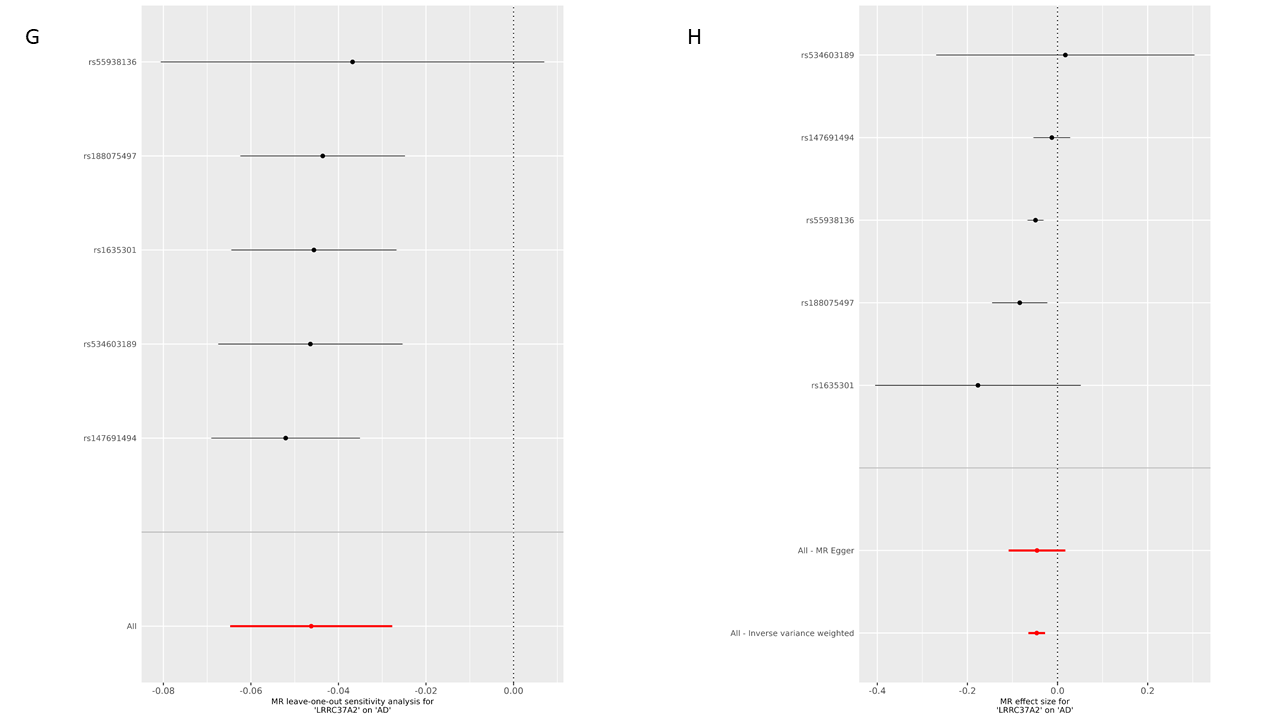


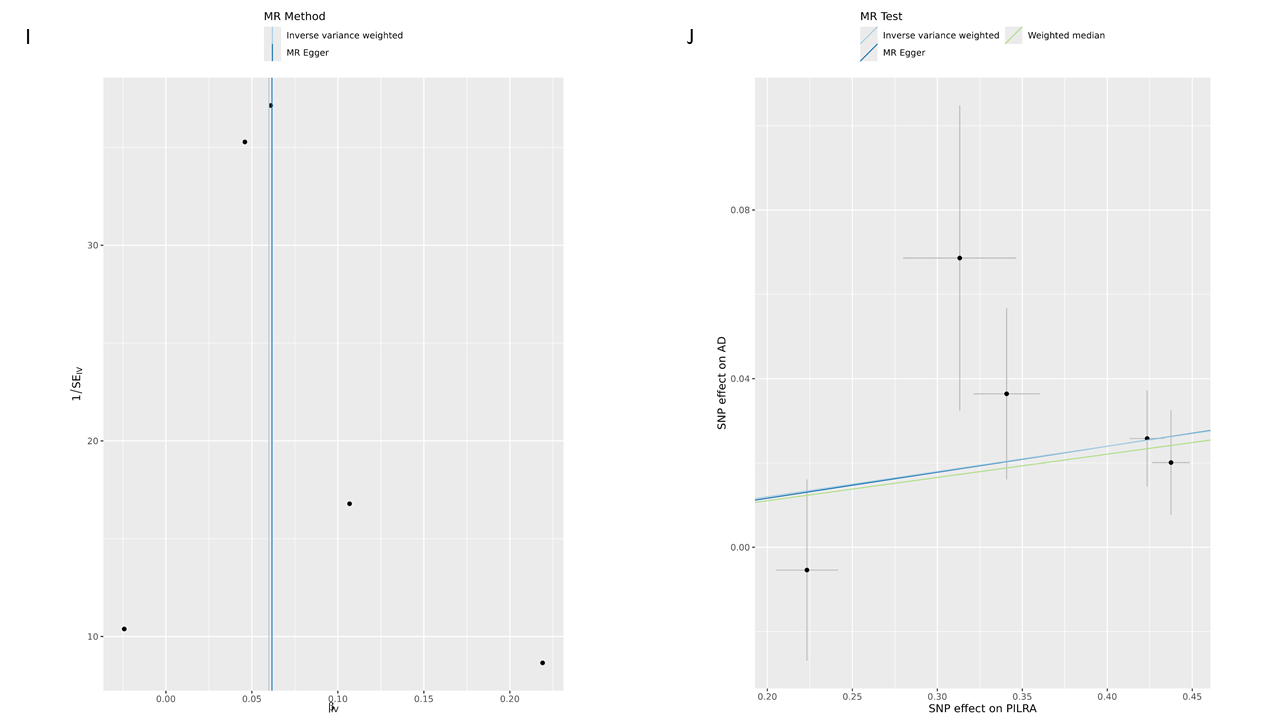


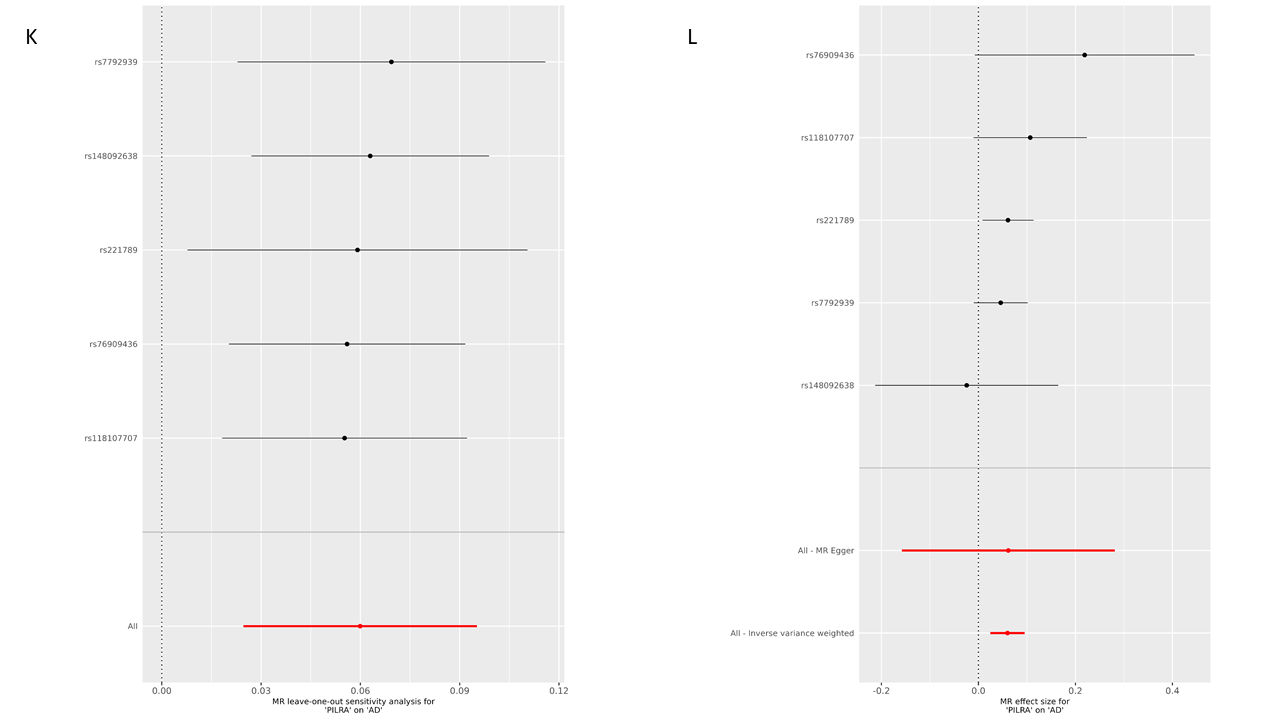


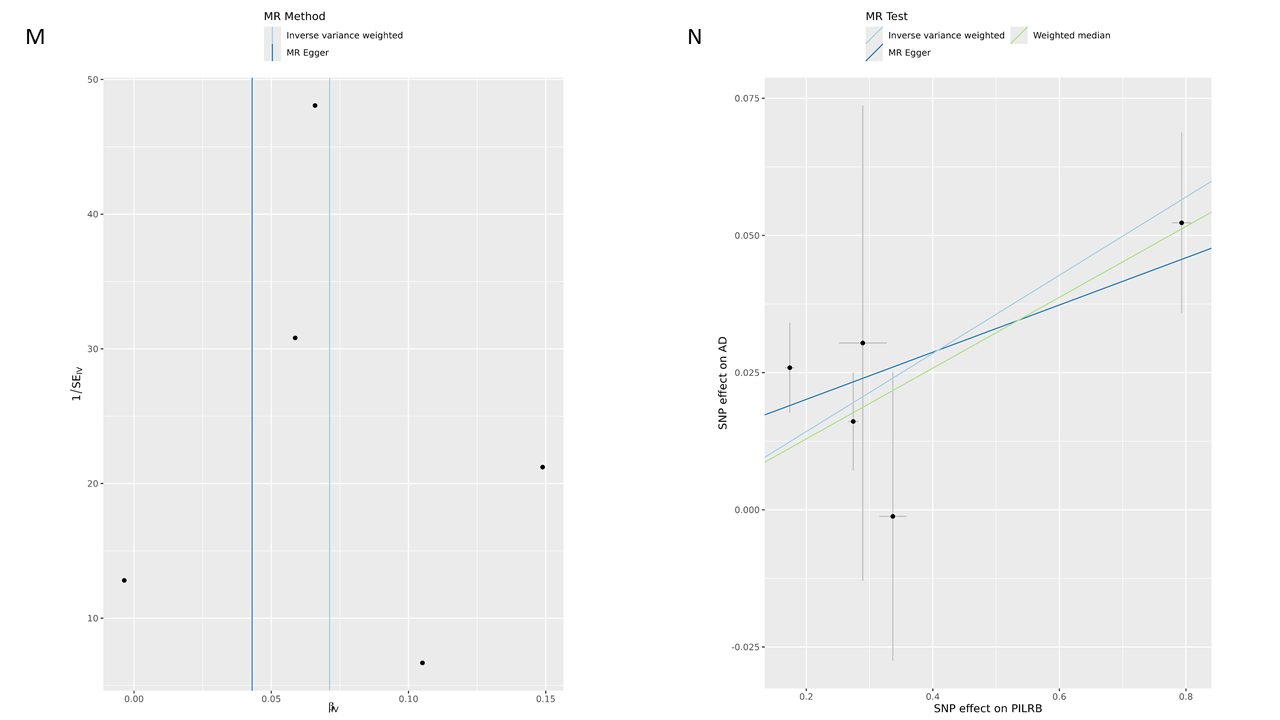


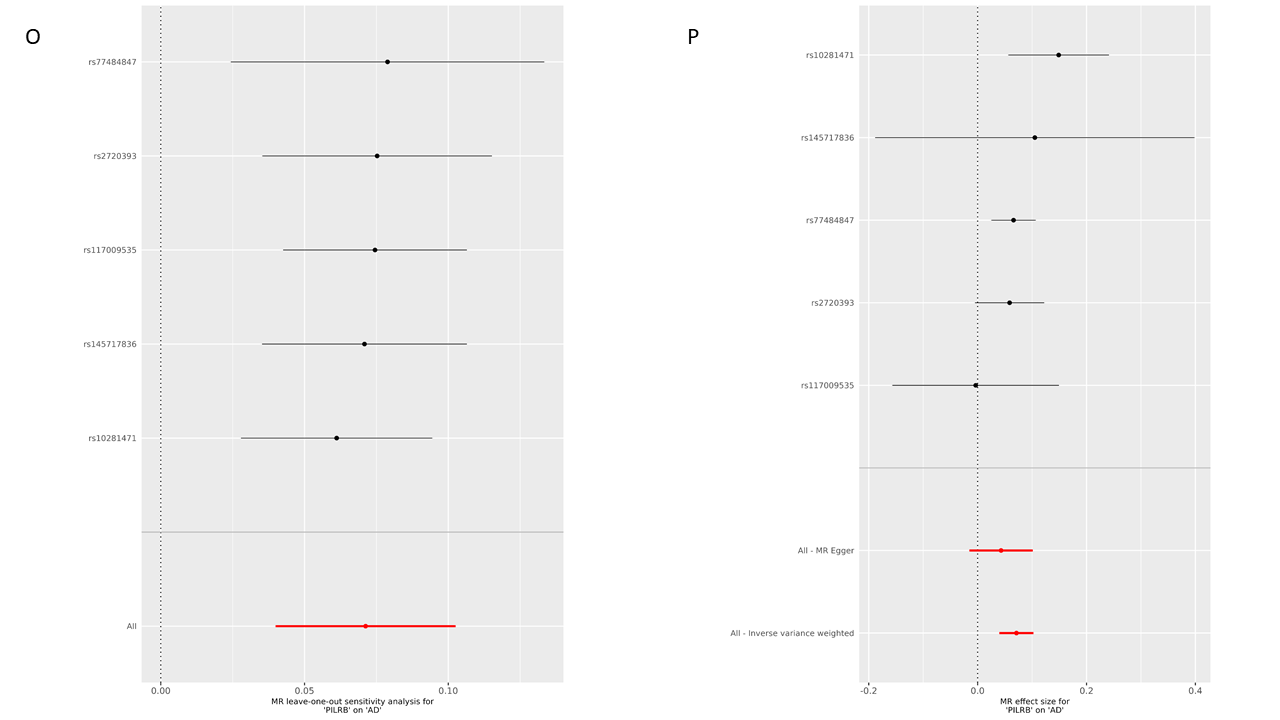


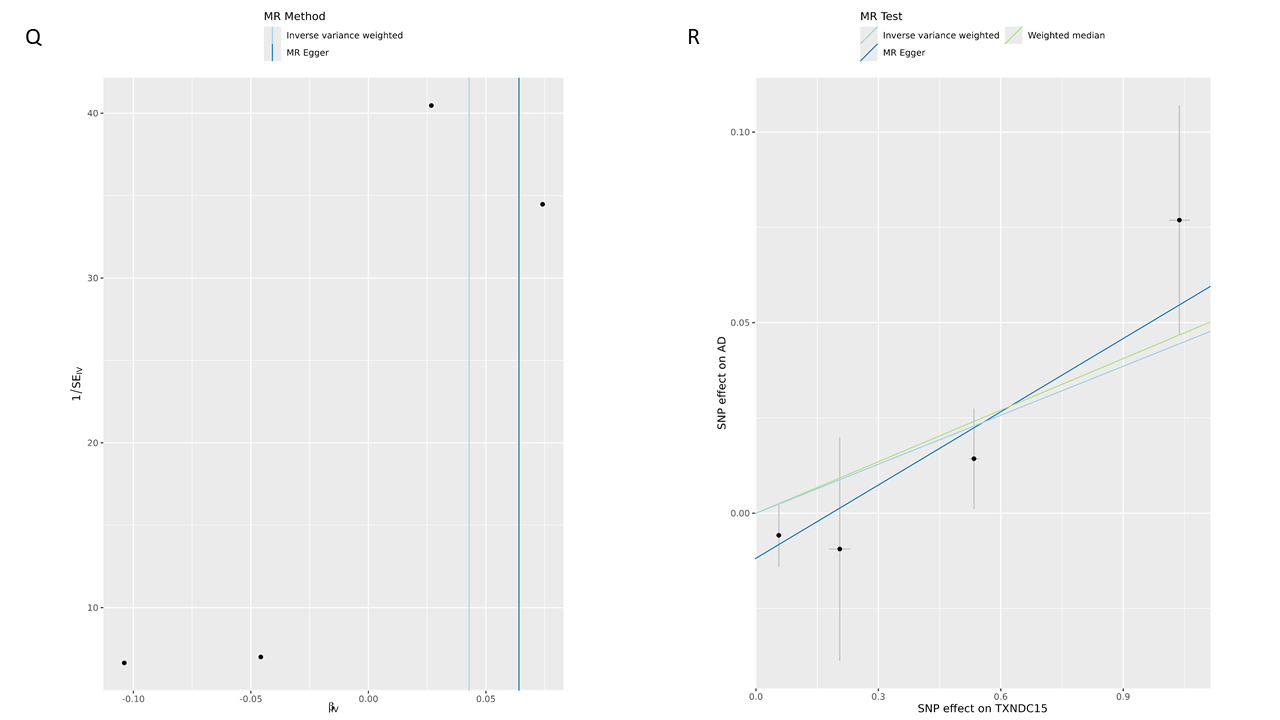


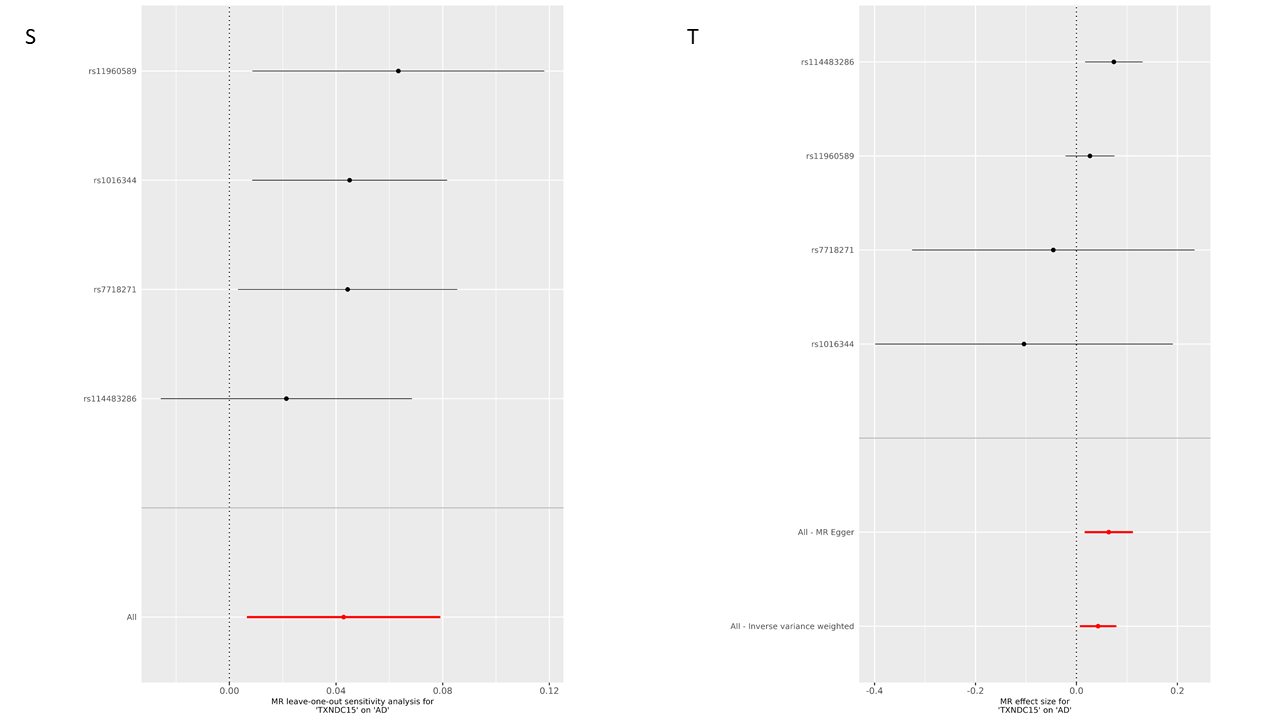


**Figure S1. Plots of five identified MR associations in discovery stage.** Funnel plot (**A**), scatter plot (**B**), leave-one-out plot (**C**) and forest plot (**D**) for MR association of protein LILRB2 on AD. Funnel plot (**E**), scatter plot (**F**), leave-one-out plot (**G**) and forest plot (**H**) for MR association of protein LRRC37A2 on AD. Funnel plot (**I**), scatter plot (**J**), leave-one-out plot (**K**) and forest plot (**L**) for MR association of protein PILRA on AD. Funnel plot (**M**), scatter plot (**N**), leave-one-out plot (**O**) and forest plot (**P**) for MR association of protein PILRB on AD. Funnel plot (**Q**), scatter plot (**R**), leave-one-out plot (**S**) and forest plot (**T**) for MR association of protein TXNDC15 on AD.


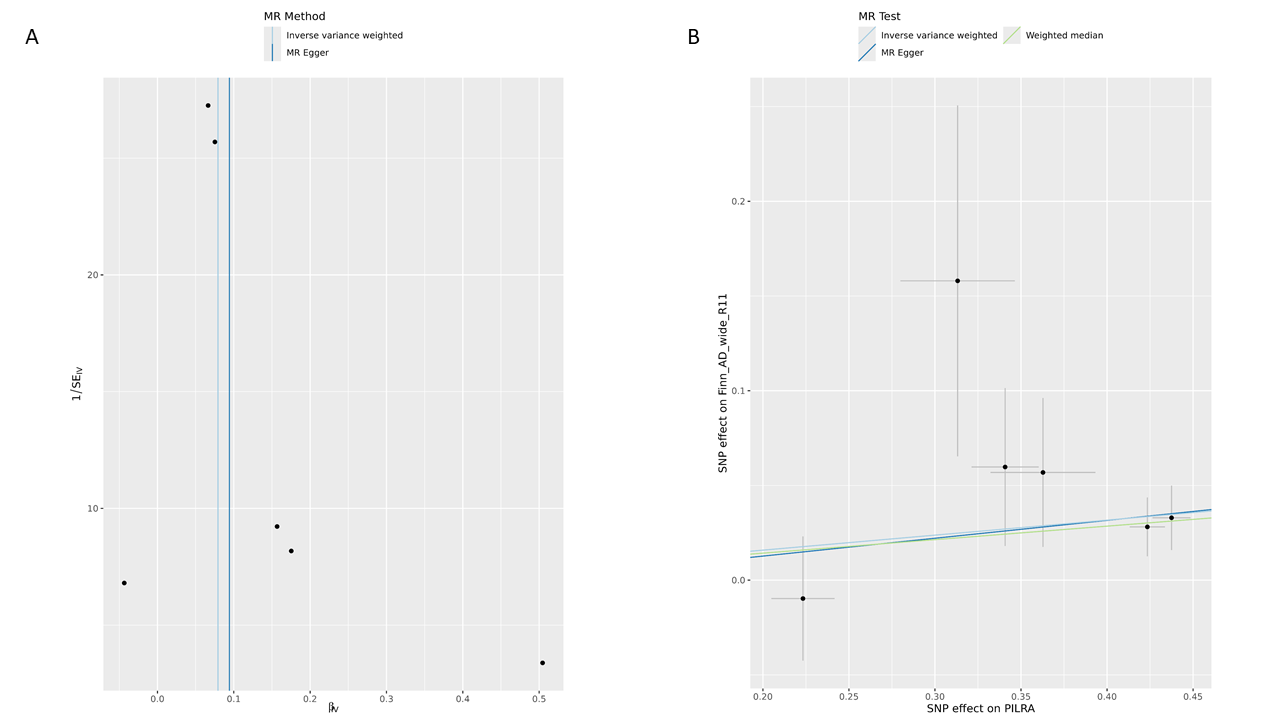


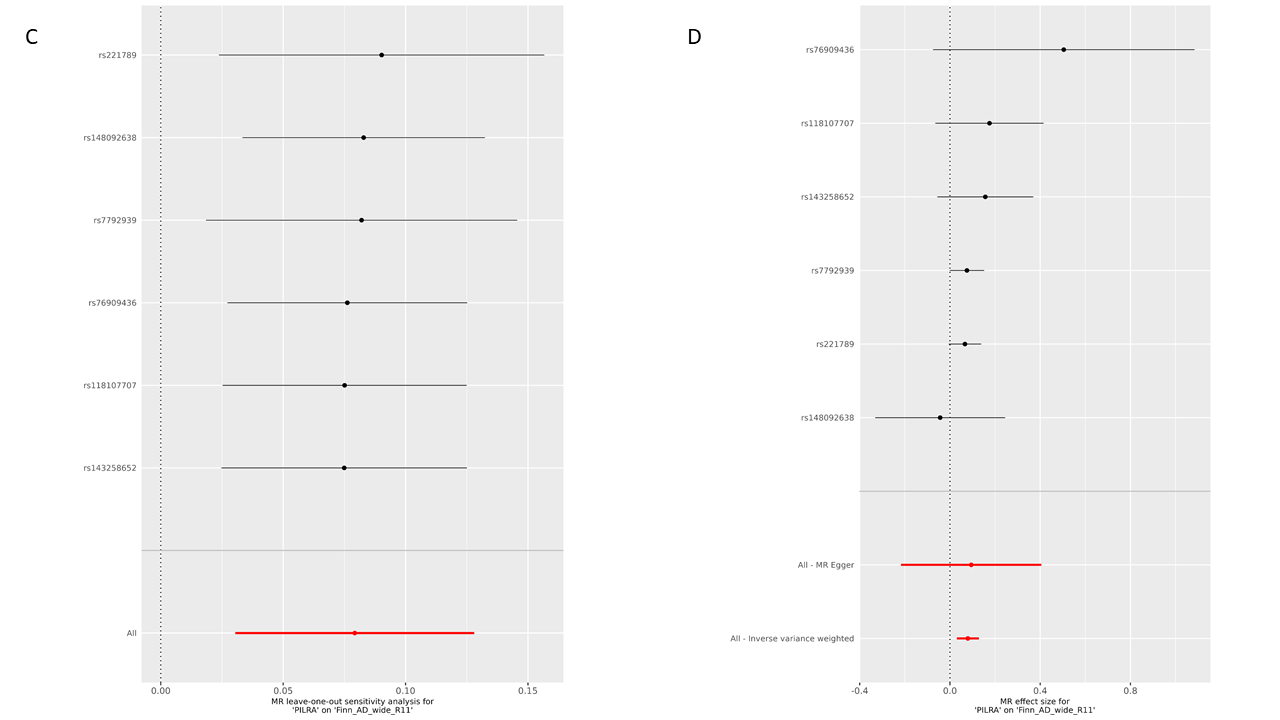


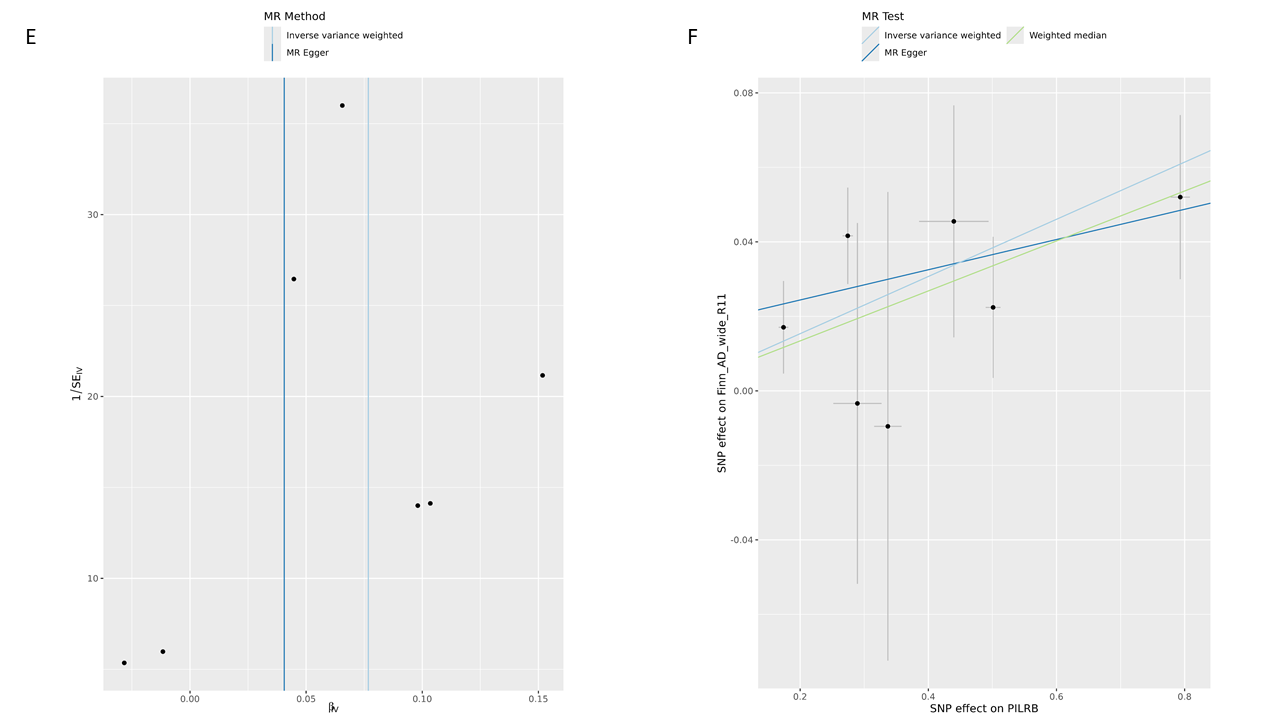


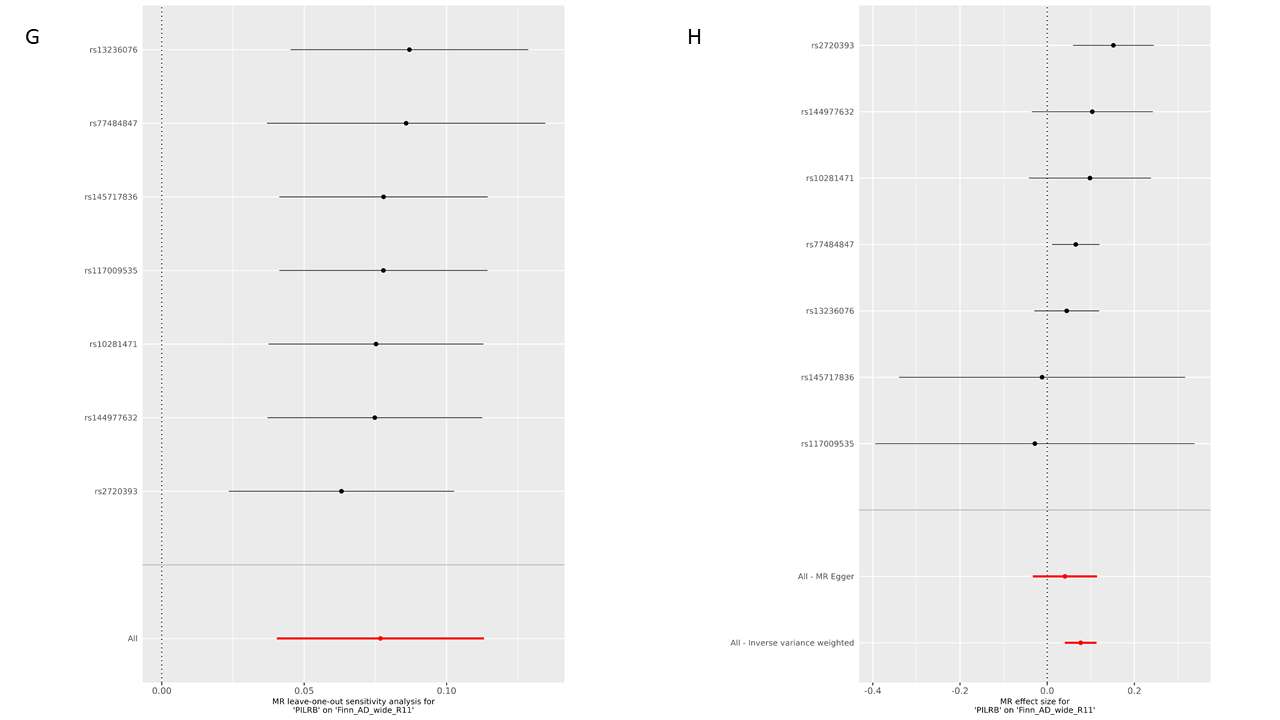


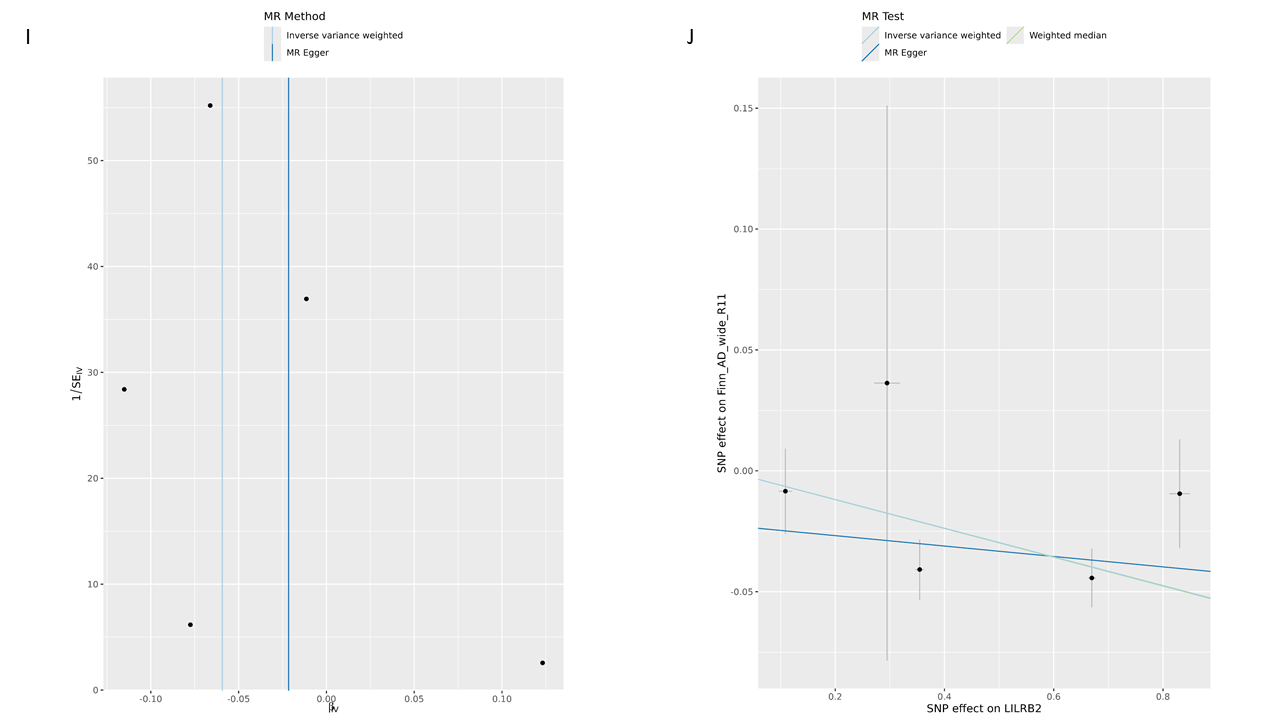


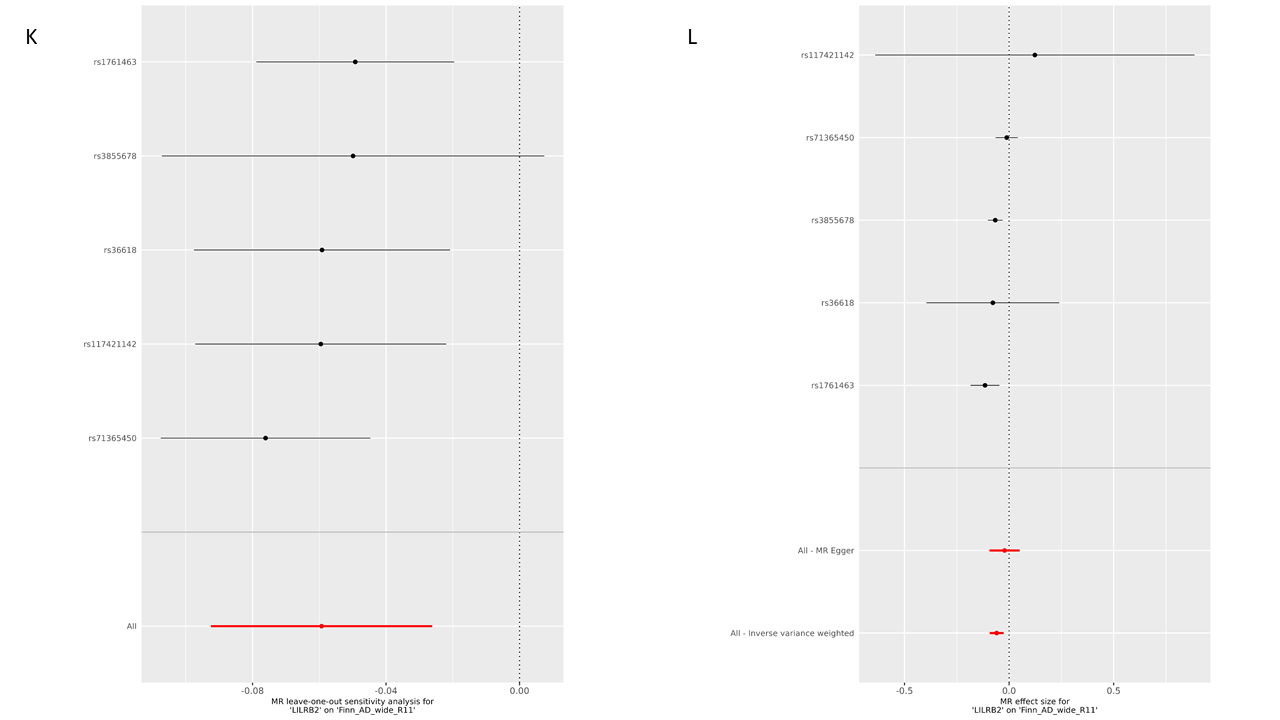


**Figure S2. Plots of three validated MR associations in replication stage.** Funnel plot (**A**), scatter plot (**B**), leave-one-out plot (**C**) and forest plot (**D**) for MR association of protein PILRA on AD. Funnel plot (**E**), scatter plot (**F**), leave-one-out plot (**G**) and forest plot (**H**) for MR association of protein PILRB on AD. Funnel plot (**I**), scatter plot (**J**), leave-one-out plot (**K**) and forest plot (**L**) for MR association of protein LILRB2 on AD.


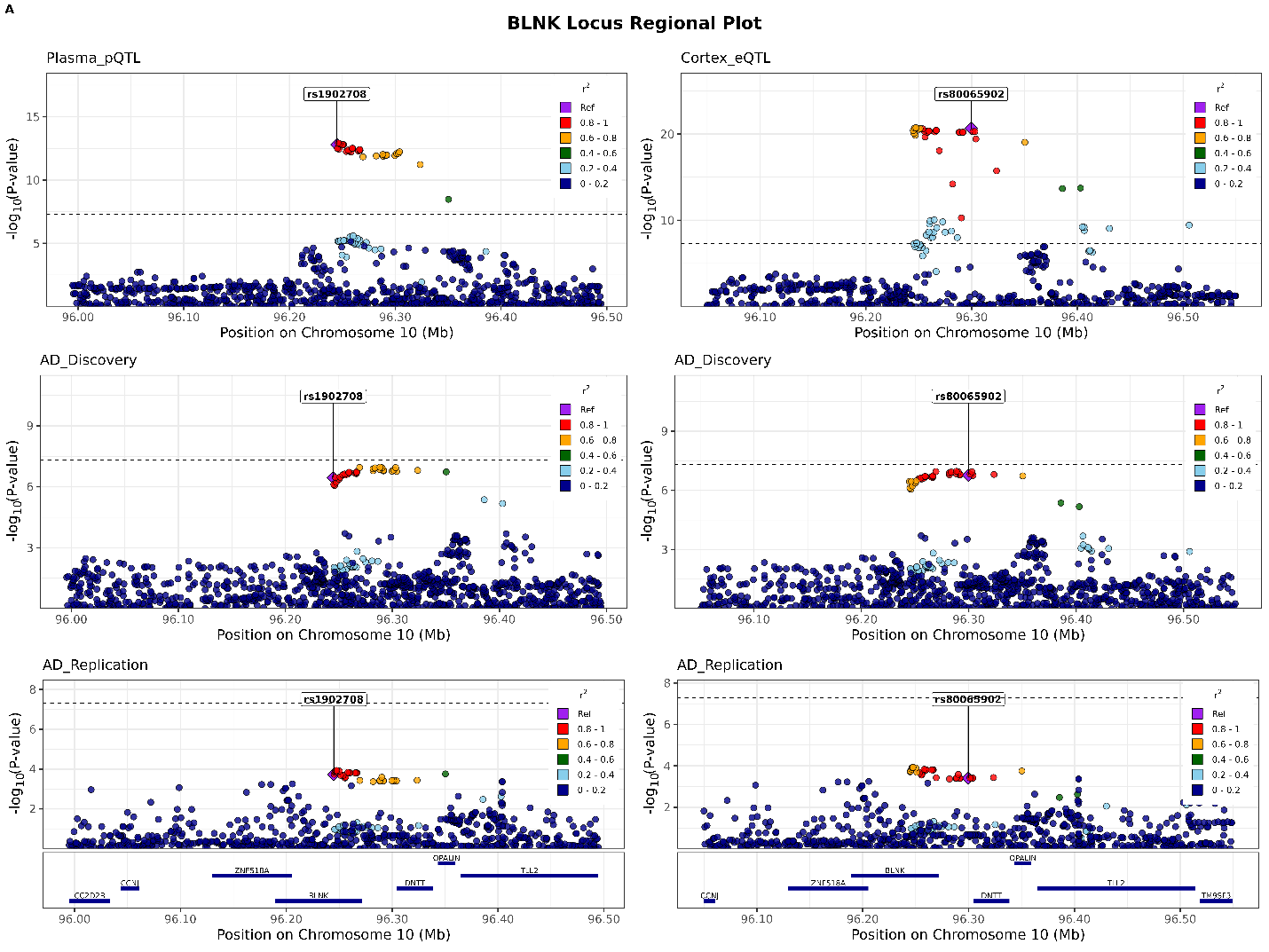


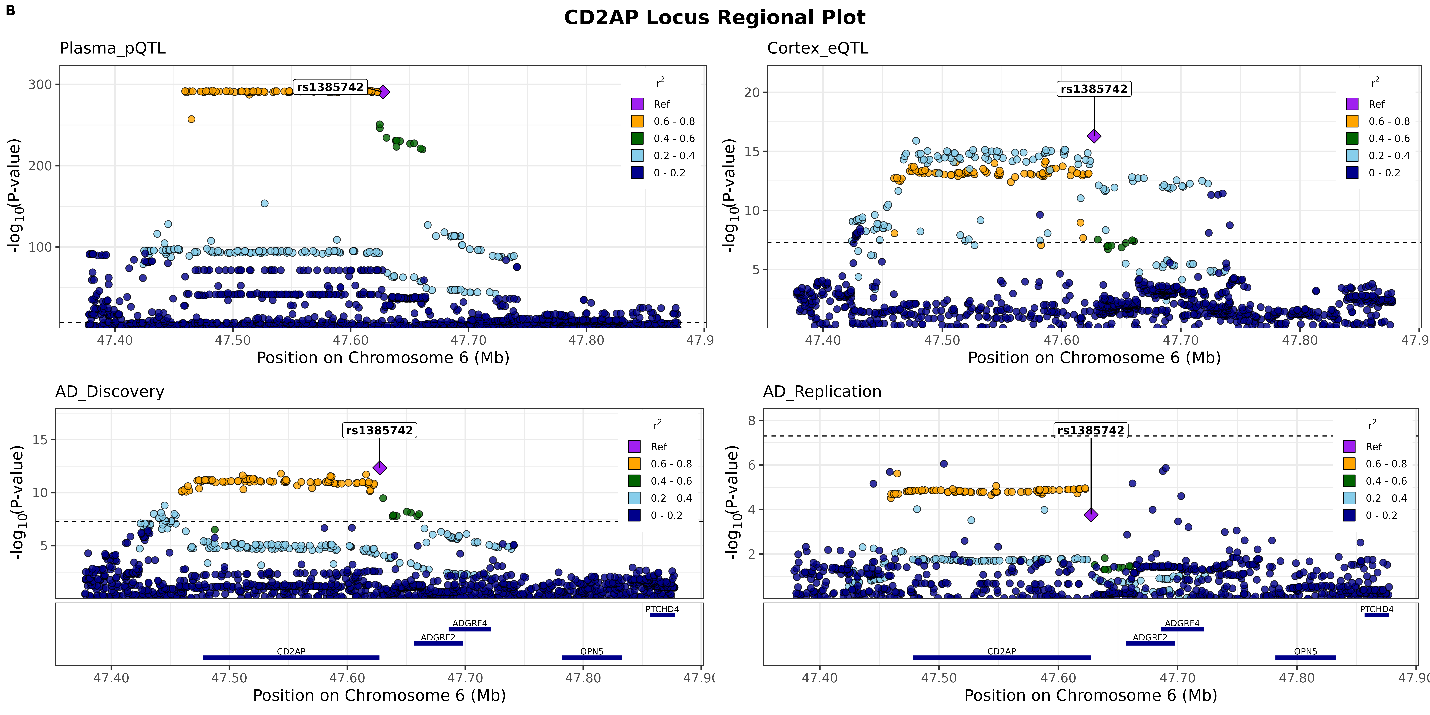


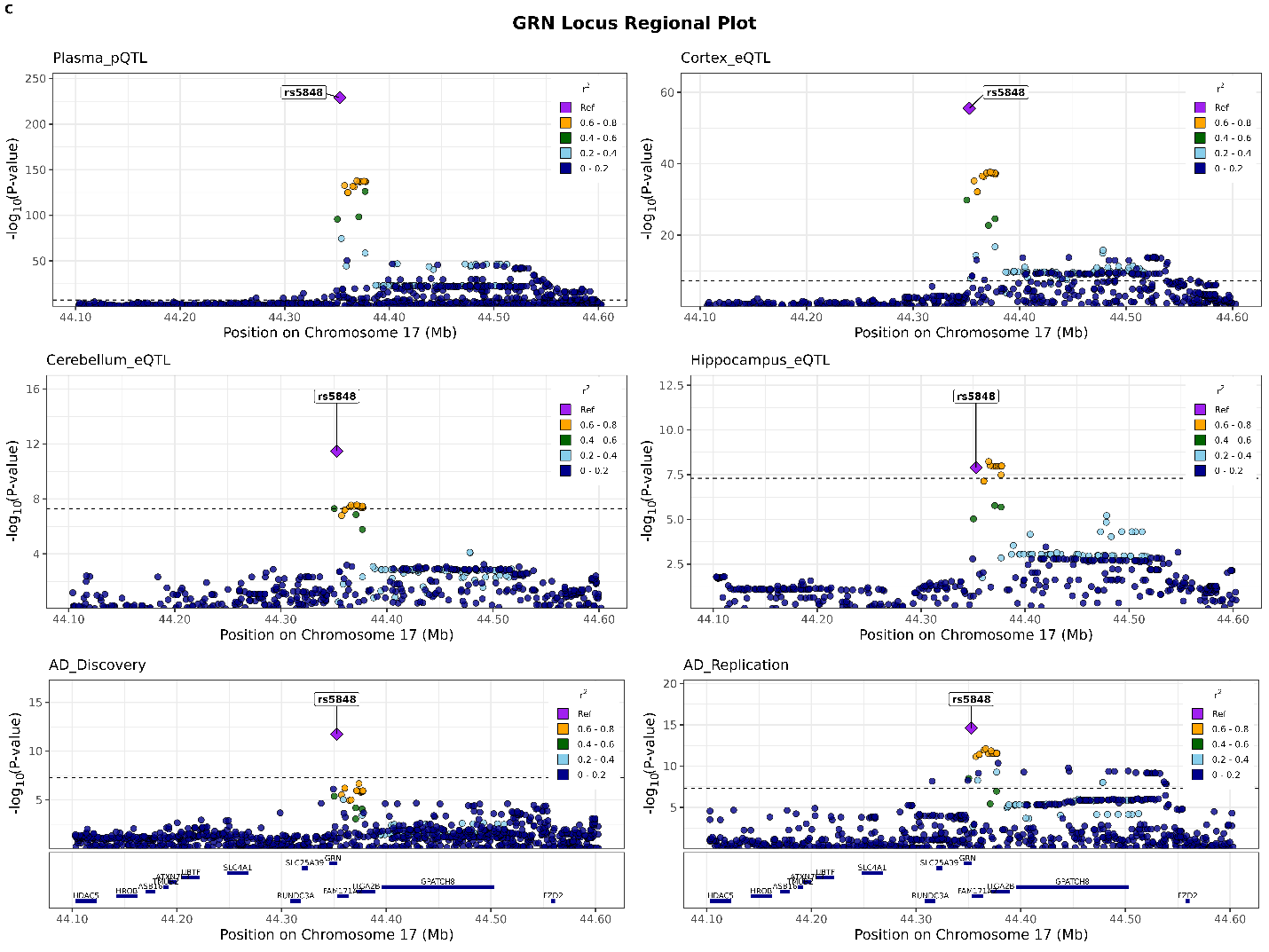


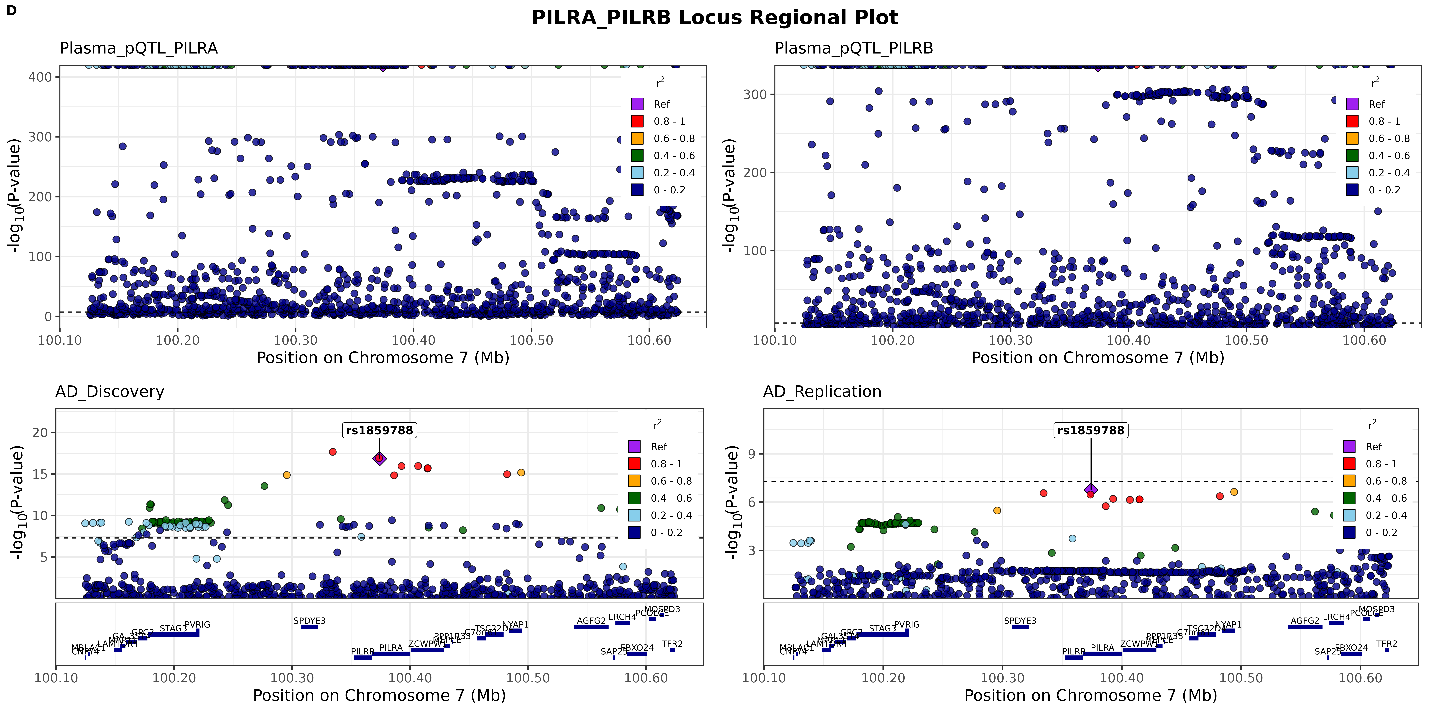


**Figure S3. Regional association plots for 5 putative plasma protein colocalization results.** **A**, BLNK Locus Regional Plot. The colocalized plasma *cis*-pQTL SNP rs1902708 and cortex *cis*-eQTL SNP rs80065902 are marked in purple. **B**, CD2AP Locus Regional Plot. The colocalized SNP rs1385742 in plasma *cis*-pQTL and cortex *cis*-eQTL are marked in purple. **C**, GRN Locus Regional Plot. The colocalized SNP rs5848 in plasma *cis*-pQTL, cortex *cis*-eQTL, cerebellum *cis*-eQTL and hippocampus *cis*-eQTL are marked in purple. **D**. PILRA_PILRB Locus Regional Plot. The colocalized SNP rs1859788 is not able shown in PILRA and PILRB plasma *cis*-pQTL due to the extreme small p value.
